# Investigation of fragmentation behaviours of isoquinoline alkaloids by mass spectrometry combined with computational chemistry

**DOI:** 10.1038/s41598-019-57406-7

**Published:** 2020-01-20

**Authors:** Zhixing Qing, Yuqin Xu, Liuyi Yu, Jinghong Liu, Xiuqiong Huang, Zhaoshan Tang, Pi Cheng, Jianguo Zeng

**Affiliations:** 1 Hunan Key Laboratory of Traditional Chinese Veterinary Medicine, Hunan Agricultural University, Changsha, 410128 China; 2 College of Food Science and Technology, Hunan Agricultural University, Changsha, 410128 China; 3 School of pharmacy, Hunan University of Chinese Medicine, Changsha, 410208 China; 4 Department of pharmacy, First Affiliated Hospital of Hunan University of Chinese Medicine, Changsha, 410007 China; 5 Micolta Bioresource Inc., Changsha, 410005 China

**Keywords:** Chemistry, Energy science and technology, Mathematics and computing

## Abstract

Isoquinoline alkaloids, which are one of the most important types of alkaloids, are extensively distributed in herbal medicines. However, systematic and comprehensive investigations of the fragmentation behaviours of isoquinoline alkaloids have rarely been reported. Therefore, the goal of the present study is to simultaneously investigate the collision-induced dissociation patterns and the corresponding mechanism of isoquinoline alkaloids by mass spectrometry (MS) combined with computations. Nineteen types of isoquinoline alkaloids (66 compounds) were used as references to identify the characteristic fragmentation behaviours by quadrupole time-of-flight mass spectrometry (Q-TOF/MS) in positive electrospray ionization (ESI) mode. These types of isoquinoline alkaloids were divided into three categories primarily by the characteristic [M-NHR_1_R_2_]^+^ (R_1_ and R_2_ represent the substituent groups of the *N*-atom) fragment ions. High- and low-abundance [M-NHR_1_R_2_]^+^ ions were observed respectively for type I (**1**–**13**) and type II (**14**–**29**) alkaloids, respectively; however, the characteristic fragments were not detected for type III alkaloids (**30**–**66**) because of the existence of a *p*-π conjugated system. Each type of alkaloid was further classified by its characteristic fragmentation patterns and fragment ions. In addition, isoquinoline alkaloid with vicinal methoxy and hydroxy, vicinal methoxy, methylenedioxy, methoxy, and quaternary *N*-methyl groups could form the characteristic fragments by the loss of CH_3_OH, CH_4_, CH_2_O or CO, CH_3_ and CO, and CH_3_ moieties, respectively. The mechanisms of some interesting fragmentation behaviours, such as the formation of [M-NH_3_]^+^ and [M-CH_3_]^+^ fragment ions, were further demonstrated by computational chemistry. These characteristic fragmentation behaviours and fragment ions of isoquinoline alkaloids provide a solid foundation for the rapid and high-efficiency structural elucidation of similar metabolites in plant-derived medicines.

## Introduction

**Figure 1:**
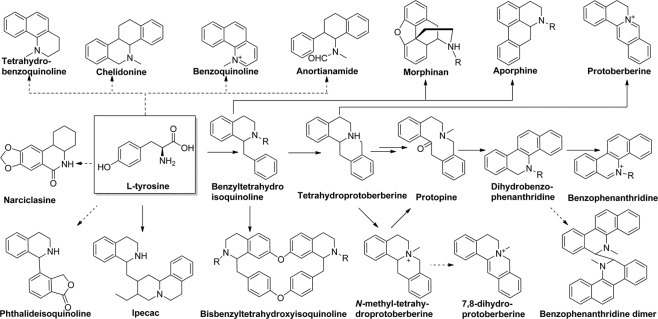
Biosynthetic pathways to 19 types of isoquinoline alkaloids (the solid arrow indicates the verification pathways, and the dashed arrow represents the proposed pathways).

Isoquinoline alkaloids, such as the analgesic agents morphine and codeine, the anticancer and antitussive drug noscapine, and the antimicrobial agents berberine and sanguinarine, are derived biosynthetically from the amino acid tyrosine in the plant kingdom (Fig. 1)^1,2^. They are distributed extensively in herbal medicines in many parts of the world^3^. Modern pharmacology studies have demonstrated that isoquinoline alkaloids have a wide range of biological activities, such as antimicrobial, antiviral, anticancer, antiproliferative, and antiplasmodial activities and acetylcholinesterase inhibitory and pain-killing effects^4,5^. To date, more than 4000 isoquinoline alkaloids have been detected from at least 10 plant families, namely, Papaveraceae, Berberidaceae, Rutaceae, Menispermaceae, Alangiaceae, Fabaceae, Ranunculaceae, Lauraceae, Annonaceae, and Fumariaceae^3^. In addition to these well-known compounds, a series of isoquinoline alkaloids, which may have potential biological activities, are still unknown. Further research to identify their structures in plant-derived medicines is necessary.

The resurgence of interest in the plant kingdom as an important source for discovering new chemotherapeutic drugs or lead compounds has prompted the urgent need for modern mass spectrometric methods that enable rapid screening and characterization of these compounds. In a specific plant, analogues with the same skeleton but different substituent groups are biosynthesized synchronously in differing amounts though specific biosynthetic pathways^6^. Since these analogues typically display similar tandem mass spectrometry (MS/MS) fragmentation patterns, investigation of the fragmentation behaviours of well-characterized references is a valid approach for determining the structure of unknown analogues^7^. However, in some previous studies^8–11^, only limited isoquinoline alkaloids were determined in plant medicine by their MS/MS data. One of the main reasons is the lack of enough reference samples for investigating the fragmentation behaviours. Therefore, in the present study, the fragmentation patterns of 19 types of isoquinoline alkaloids (66 compounds) were systematically and comprehensively investigated to provide a solid foundation for identifying analogues in plant-derived medicines.

In some previous studies, the fragmentation behaviours or characteristic fragment ions of benzyltetrahydroisoquinoline^12–15^, aporphine^10,14–16^, tetrahydroprotoberberine^8,10,12–15,17^, protopine^10,14,15^, protoberberine^8,10,11,14,15,17–19^, *N*-methyltetrahydroprotoberberine^14,15^, dihydrobenzo- phenanthridine^14,15^, benzophenanthridine^14,15,20,21^, chelidonine^22^, morphine^23^, narciclasine^24^, phthalideisoquinoline^25^, ipecac^26^, and bisbenzyltetrahydroxyisoquinoline^27^ have been investigated. However, comparative studies on the fragmentation pathways of these isoquinoline alkaloids have rarely been reported, which leads to the neglect of some interesting and characteristic fragmentation patterns. In this study, the fragmentation behaviours of 19 types of isoquinoline alkaloids were simultaneously investigated, and their fragmentation pathways were comprehensively and systematically compared. Some interesting fragmentation patterns and characteristic fragments, such as the low-abundance [M-NHR_1_R_2_]^+^ fragment ions for tetrahydroprotoberberine, *N*-tetrahydroproto- berberine, protopine, morphine, and phthalideisoquinoline-type alkaloid, were observed for the first time.

Electrospray ionization mass spectrometry (ESI-MS) is an ideal tool to study the dissociation reactions of organic compounds and has been widely applied to investigate the fragmentation pathways of isoquinoline alkaloids^14–17^. However, the fragmentation mechanism in ESI-MS has not yet been well studied. Therefore, in this present study, some important and interesting mechanisms of dissociation reactions of isoquinoline alkaloids were primarily proposed. In addition, density functional theory (DFT)-based calculations were performed to support the proposed mechanism.

## Results and Discussion

### Fragmentation behaviours of benzyltetrahydroisoquinoline, aporphine, ipecac, chelidonine and bisbenzyltetrahydroisoquinoline

**Figure 2:**
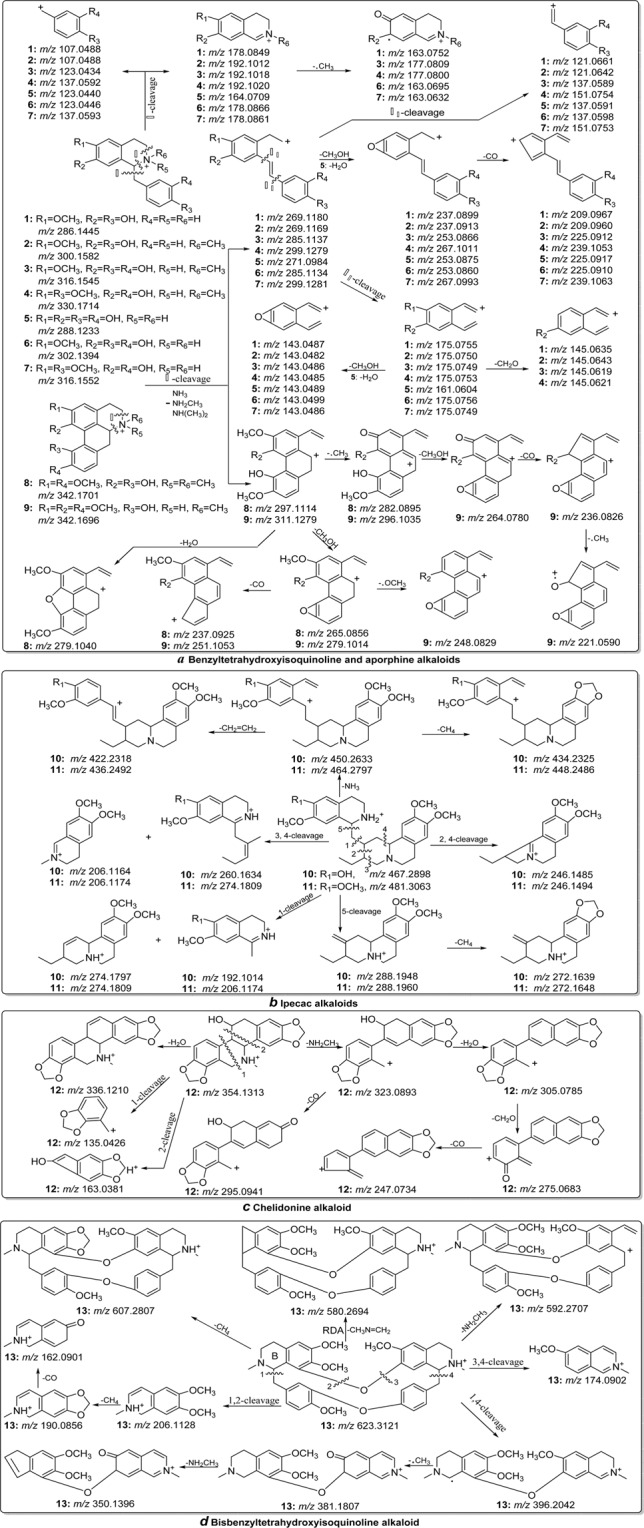
The proposed fragmentation behaviours of benzyltetrahydroisoquinoline and aporphine (**a**), ipecac (**b**), chelidonine (**c**), and bisbenzyltetrahydroisoquinoline (**d**).

In the MS/MS investigation of benzyltetrahydroisoquinoline (**1**–**7**), aporphine (**8**–**9**), ipecac (**10**–**11**), chelidonine (**12**) and bisbenzyltetrahydroisoquinoline alkaloid (**13**) (type I), the high-abundance fragments [M-45.0578]^+^ ([M-NH(CH_3_)_2_]^+^), [M-31.0422]^+^ ([M-NH_2_CH_3_]^+^) and [M-17.0265]^+^ ([M-NH_3_]^+^) were observed according to the different substituent groups on the *N*-atom. These fragment ions are attributed to the loss of neutral NHR_1_R_2_ (R_1_ and R_2_ represent the substituent groups on the *N*-atom) moiety from the protonated molecules. In the MS/MS spectra of alkaloids **1**–**13** (Fig. S1), the high- abundance ions at *m/z* 269.1180, 269.1169, 285.1137, 299.1279, 271.0984, 285.1134, 299.1281, 297.1114, 311.1279, 450.2633, 464.2797, 323.0893 and 592.2707 were detected, respectively, which corresponded to the loss of an NHR_1_R_2_ moiety from the protonated molecular weights at *m/z* 286.1445, 300.1582, 316.1545, 330.1714, 288.1233, 302.1394, 316.1552, 342.1701, 342.1696, 467.2898, 481.3063, 354.1313 and 623.3121 (Fig. 2). The characteristic fragmentation behaviours and corresponding distinctive fragment ions play a diagnostic role in the discrimination of other alkaloid types^12–16^.

**Figure 3:**
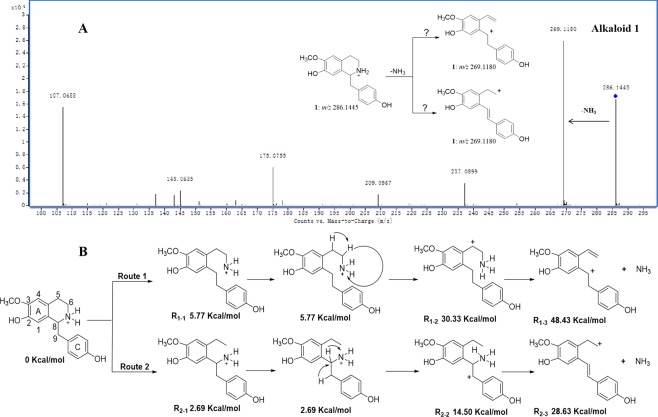
The MS/MS spectrum of alkaloid **1** (**A**) and the two proposed fragmentation mechanisms for the loss of an NH_3_ moiety (**B**).

The mechanism for the formation of [M-NHR_1_R_2_]^+^ fragment ions for type I isoquinoline alkaloids has rarely been investigated and reported. In this study, a representative compound (alkaloid **1**) was selected as a model to demonstrate the most favoured protonation site and possible fragmentation mechanism. The full-scan positive ion ESI mass spectrum of alkaloid **1** displayed a protonated molecule at *m/z* 284.1445, which subsequently gave the highly abundant product ion at *m/z* 269.1180 by collision-induced dissociation (Fig. 3A). The structure contains different protonation sites, such as nitrogen atom, phenyl rings A and C, and oxygen atoms of methoxy and hydroxy groups. Each possible protonation site of alkaloid **1** was optimized at the RB3LYP/6-31 G(*d*) level. The relative energy of these structures was calculated, as shown in Table 1. The computational results indicated that nitrogen is the most favoured protonation site because the protonated molecule has the lowest energy, which agrees with previous reports^14–17^.

**Table 1:** Relative energies of structures with different protonation sites of alkaloid **1**.

Structure	Site of protonation	Relative energy (kcal/mol)
	Nitrogen	0.0
	Phenyl ring A	24.77
	Phenyl ring C	34.19
	Oxygen of 3-OCH_3_	47.69
	Oxygen of 2-OH	52.55
	Oxygen of 4′-OH	60.84

The neutral loss of NH_3_ from the protonated molecule at *m/z* 284.1445 was triggered by the arrival of a proton, which is described as a so-called dissociative protonated site. Two possible fragmentation mechanisms (Fig. 3B) were proposed for the competitive proton transfer reactions to explain the formation of the molecular ion peak at *m/z* 269.1180. In pathway 1, cleavage of the N-C_8_ bond by proton-induced dissociation leads to formation of the active intermediate (**R**_**1-1**_) first. Then, the hydrogen atom transfers from C-5 to the *N*-atom through a three-membered-ring transition state to form **R**_**1-2**_. Finally, the neutral loss of NH_3_ was observed by cleavage of the bond between C-6 and the *N*-atom and formation of the fragment ion (**R**_**1-3**_). In pathway 2, cleavage of the N-C_6_ bond leads to formation of the active intermediate (**R**_**2-1**_) first. Then, the hydrogen atom migrates from C-9 to the *N*-atom formation in the transition state **R**_**2-2**_. Finally, the neutral loss of NH_3_, which gave the fragment ion (**R**_**2-3**_), was also detected through the cleavage of the bond between C-8 and the *N*-atom. It is difficult to deduce whether pathway 1 or 2 is the more reasonable fragment pathway without theoretical calculations. The relative energies of **R**_**1-1**_, **R**_**1-2**_, and **R**_**1-3**_ are 5.77, 30.33 and 48.43 kcal/mol, which are 3.08, 15.83 and 19.80 kcal/mol higher than those of **R**_**2-1**,_ **R**_**2-2**_, and **R**_**2-3**_, respectively. These results suggest that pathway 2 is the more reasonable fragmentation mechanism for the neutral loss of NH_3_ because pathway 1 needs to overcome a higher energy barrier. The schematic potential energy surface for the proposed fragmentation mechanism is given in Fig. S2 to quantitatively describe the energy requirement of the reaction.

In addition to the above characteristic fragmentation pattern, several common fragmentation behaviours were observed for these five types of isoquinoline alkaloids. Alkaloids with vicinal methoxy and hydroxy groups could produce the characteristic fragments by the neutral loss of a CH_3_OH molecule. In the MS/MS spectra of alkaloids **1**, **2**, **3**, **4**, **6**, **7**, **8** and **9** (Fig. S1), the fragment ions at *m/z* 175.0755, 175.0750, 175.0749, 175.0753, 175.0756, 175.0749, 265.0856 and 279.1014 corresponded to the loss of a CH_3_OH moiety from their mother fragment ion (Fig. 2(a)). Alkaloids with vicinal methoxy groups could produce the fragment ions by the loss of a CH_4_ moiety. The fragments at *m/z* 434.2325, 448.2486 and 607.2807 for compounds **10**, **11** and **13** were generated by the loss of a CH_4_ molecule from the ions at *m/z* 450.2633, 464.2797 and 623.3121 (Fig. 2(b,d)) respectively. Alkaloids with methylenedioxy groups could produce some product ions by loss of a CO or CH_2_O moiety from their precursor ions. In the MS/MS spectrum of alkaloid **12** (Fig. S1), the molecular ion peak at *m/z* 275.0683 was observed, corresponding to loss of a CH_2_O moiety from the precursor ion at *m/z* 305.0785, and subsequent loss of a CO and CH_2_O moiety leading to formation of fragment ions at *m/z* 247.0737 and 217.0627 was also detected (Fig. 2(c))^14,15,22,26,27^.

**Figure 4:**
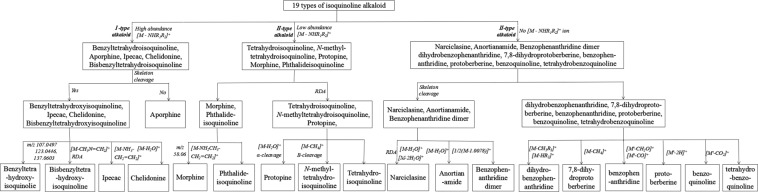
The proposed flowchart for categorization of 19 types of isoquinoline alkaloid.

Benzyltetrahydroisoquinoline, aporphine, ipecac, chelidonine and bisbenzyltetrahydro- isoquinoline alkaloid could be well categorized by their characteristic fragmentation patterns. The MS/MS spectra of benzyltetrahydroisoquinoline, ipecac, chelidonine and bisbenzyltetrahydro- isoquinoline alkaloid show a wide spectral range because of the cleavage of these alkaloid skeletons. However, the MS/MS spectra of aporphine alkaloids (compounds **8** and **9**, Fig. S1) was different from the above four alkaloid types because of the conjugated structure. Characteristic fragments below *m/z* 200 were not observed, and the main product ions were formed by the loss of substituent groups (Fig. S1)^14–16^. From the ESI-MS/MS spectra of benzyltetrahydroisoquinoline alkaloids (**1**–**7**), a series of fragment ions at *m/z* 107.0448, 107.0448, 123.0434, 137.0592, 123.0440, 123.0446 and 137.0593 were detected, which corresponded to β-cleavage of the skeleton (Fig. 2(a)). These characteristic ions play an important role in the discrimination of the other three types^12–15^. The fragmentation behaviours of bisbenzyltetrahydroisoquinoline were different from those of benzyltetrahydroisoquinoline alkaloids. The above characteristic fragment ions were not observed in the MS/MS spectrum of alkaloid **13** (Fig. S1). However, the fragment at *m/z* 580.2694 ([M-CH_3_N = CH_2_]^+^) was formed by retro-Diels-Alder (RDA) fragmentation at the B-ring of the protonated molecule at *m/z* 623.3121 (Fig. 2(d)). The fragmentation pathway was regarded as a characteristic marker for bisbenzyltetrahydroisoquinoline alkaloids^27^. In the MS/MS spectra of alkaloids **10** and **11**, most of the fragment ions were produced by cleavage of the alkaloid skeleton. However, the characteristic ions at *m/z* 422.2318 and 436.2492 were formed by successive loss of NH_3_ and CH_2_ = CH_2_ moieties from the protonated molecular ion at *m/z* 467.2898 and 481.3063, respectively, which were regarded as a diagnostic fragmentation pattern for ipecac-type alkaloids(Fig. 2(b))^26^. The MS/MS spectra of chelidonine alkaloids can be well distinguished from other alkaloid types by the presence of [M-H_2_O]^+^ ions. In the MS/MS spectrum of alkaloid **12** (Fig. S1), the ion at *m/z* 336.1210 was formed by the neutral loss of a H_2_O molecule from the protonated molecular ion at *m/z* 354.1313^22^. The proposed flowchart for identification of each alkaloid type is shown in Fig. 4. The proposed fragmentation behaviours of benzyltetrahydro- isoquinoline, aporphine, ipecac, chelidonine and bisbenzyltetrahydroisoquinoline alkaloid are shown in Fig. 2^10,12–16,22,26,27^.

### Fragmentation behaviours of tetrahydroprotoberberine, *N*-methyltetrahydroprotoberberine, protopine, morphinan and phthalideisoquinoline

**Figure 5:**
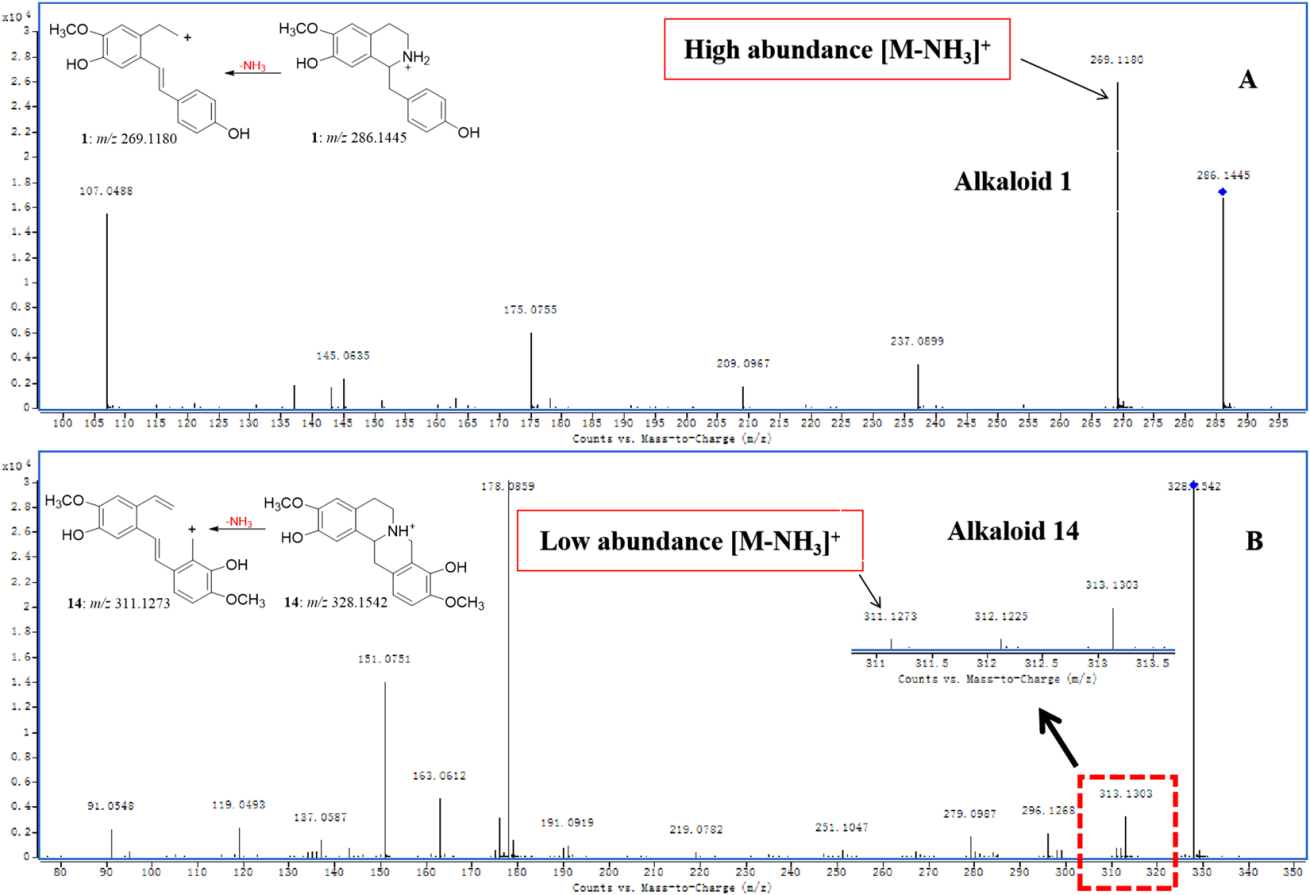
The MS/MS spectra of alkaloids **1** (**A**) and **14** (**B**) and the corresponding [M-NH_3_]^+^ fragment ion.

In the MS/MS spectra of tetrahydroprotoberberine, *N*-methyltetrahydroprotoberberine, protopine, morphinan and phthalideisoquinoline alkaloid (type II), a series of low-abundance fragment ions (*m/z* 311.1273, 309.1121, 307.0987, 325.1467, 323.1290, 339.1601, 307.0952, 309.1121, 311.1284, 323.1287, 311.1280, 323.0898, 339.1179, 255.1010, 269.1306 and 383.1143 for alkaloids **14**–**29**, respectively) were detected, which were rarely reported in previous studies. These fragment ions were generated by the loss of the NHR_1_R_2_ moiety from the protonated molecules (Fig. S1). These low- abundance fragments play a diagnostic role in distinguishing between type I alkaloids, which always produced a high-abundance ([M-NHR_1_R_2_]^+^) ion in their MS/MS spectra. Taking alkaloids **1** and **14** as examples, the high-abundance [M-NH_3_]^+^ ion at *m/z* 269.1180 (ppm 2.97), which was easily observed in the MS/MS spectrum of alkaloid **1** (Fig. 5), was produced by the loss of an NH_3_ moiety from the protonated molecule at *m/z* 286.1445 (ppm 2.44)^15,17^. However, the low-abundance [M-NH_3_]^+^ fragment at *m/z* 311.1273 (ppm −1.60), which was difficult to detect in the MS/MS spectrum of alkaloid **14** (Fig. 5), was also formed by the loss of an NH_3_ moiety from the protonated molecule at *m/z* 328.1542 (ppm −0.30).

**Figure 6:**
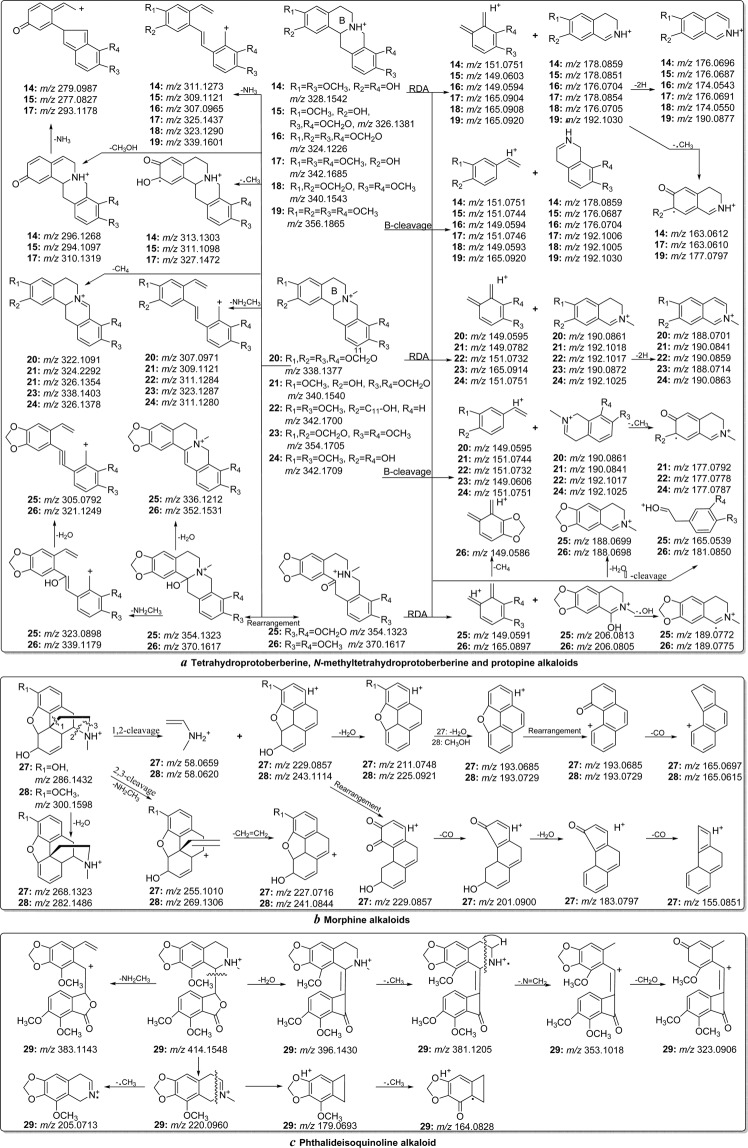
The proposed fragmentation behaviours of tetrahydroprotoberberine, *N*-methyltetrahydroprotoberberine and protopine (**a**), morphinan (**b**), and phthalideisoquinoline (**c**).

In the MS/MS spectra of tetrahydroprotoberberine, *N*-methyltetrahydroprotoberberine, and protopine, the characteristic fragment ions mainly appear below *m/z* 230, which corresponds to RDA reaction, and are regarded as diagnostic fragmentation behaviour to discriminate morphinan and phthalideisoquinoline-type alkaloids^15^. The unique fragments of protopine-type alkaloids were formed mainly by the neutral loss of H_2_O from the [M + H]^+^ ion (*e.g*, *m/z* 336.1212 and 352.1531 for alkaloids **25** and **26**, respectively) and α-cleavage of the skeleton (*e.g*, *m/z* 165.0539 and 181.0850 for alkaloids **25** and **26**, respectively) (Fig. 6(a)). These characteristic fragmentation patterns distinguish protopine from tetrahydroprotoberberine and *N*-methyltetrahydroprotoberberine^10,14,15^. Tetrahydro-protoberberine showed fragmentation pathways similar to *N*-methyltetrahydroprotoberberine. However, low- abundance [M-CH_4_]^+^ ions (*m/z* 322.1091, 324.2292, 326.1354, 338.1493 and 326.1378 for alkaloids **20**–**24**, respectively) were observed for *N*-methyltetrahydroprotoberberine alkaloids, which were formed by loss of the *N*-methyl and vicinal hydrogen atom from the protonated molecules. In addition, the B-ring cleavage reaction (forming fragments at *m/z* 176.0704 and 190.0861 for alkaloids **16** and **20**, respectively) played an important role in determining the substituent group on the *N*-atom (Fig. 6(a)). Therefore, the tetrahydroprotoberberine and *N*-methyltetrahydroprotoberberine alkaloids could be categorized by the presence of [M-CH_4_]^+^ ions and B-ring cleavage reactions^14,15^. In the MS/MS spectra of morphine and phthalideisoquinoline-type alkaloids, the fragments were formed mainly by the loss of some substituent groups and cleavage of the alkaloid skeleton (Fig. 6(b,c)). Fragment ions at *m/z* 227.0716 and 241.0844 were observed for alkaloids **27** and **28**, respectively, by the loss of NH_2_CH_3_ and CH_2_ = CH_2_ moieties from the protonated structure. The ion at *m/z* 58.06 was observed for both alkaloids, corresponding to the 1,2-cleavage reaction (Fig. 6(b)). The morphine and phthalideisoquinoline-type alkaloids could be characterized by these characteristic ions and fragmentation behaviours^23^. The proposed flowchart for identification of these five alkaloid types is shown in Fig. 4. The proposed fragmentation behaviours of tetrahydroprotoberberine, *N*-methyltetrahydroprotoberberine, protopine, morphinan and phthalideisoquinoline alkaloid are shown in Fig. 6^8,10,12–15,17,23,25^.

### Fragmentation behaviours of narciclasine, anortianamide, benzophenanthridine dimer, dihydrobenzophenanthridine, 7,8-dihydroprotoberberine, protoberberine, benzophenanthridine, benzoquinoline and tetrahydrobenzoquinoline

**Figure 7:**
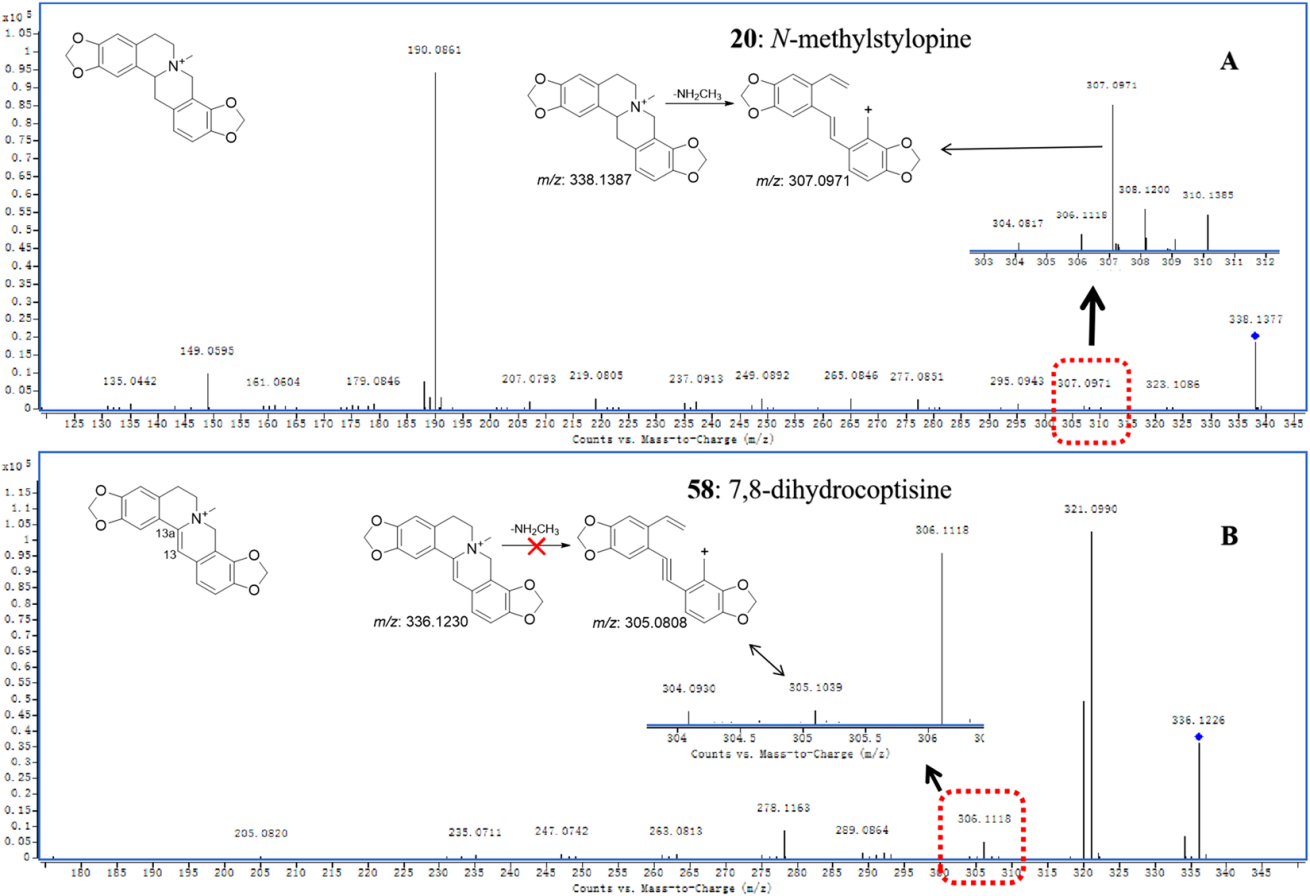
The MS/MS spectra of alkaloids **20** (**A**) and **58** (**B**).

In the MS/MS spectra of narciclasine, anortianamide, benzophenanthridine dimer, dihydrobenzophenanthridine, 7,8-dihydroprotoberberine, protoberberine, benzophenanthridine, benzoquinoline and tetrahydrobenzoquinoline alkaloid, the characteristic [M-NHR_1_R_2_]^+^ fragment ions could not be detected due to the existence of a *p*-π conjugated system in their structure. This result was obtained by comparing the MS/MS spectra of alkaloids **1**–**29** with those of alkaloids **30**–**66** (type III) on the basis of their structural characteristics. Taking alkaloids **20** and **58** as examples (Fig. 7), the low- abundance characteristic ion at *m/z* 307.0971 (ppm 0) was observed in the MS/MS spectrum of alkaloid **20**, corresponding to the loss of a NH_2_CH_3_ radical (R_1_ = H, R_2_ = CH_3_) from the protonated molecule at *m/z* 338.1377 (ppm −2.95). However, in the MS/MS spectrum of alkaloid **58**, the [M-NH_2_CH_3_]^+^ fragment ion was not detected (a suspected [M-NH_2_CH_3_]^+^ fragment at *m/z* 305.1039 was observed; however, its reached 75.7 ppm). The difference between the two structure types is that alkaloid **58** has a double bond between C-13 and C-13a and form the *p*-π conjugated system with the *N*-moiety (Fig. 7). It is difficult to escape the conclusion that the existence of a *p*-π conjugated system could lead to the absence of a characteristic [M-NHR_1_R_2_]^+^ fragment ion. The missing [M-NHR_1_R_2_]^+^ fragments played a diagnostic role in the discrimination of type I and II alkaloids (Fig. 4). The absence of [M-NHR_1_R_2_]^+^ fragment ions of type III alkaloids were observed, and corresponding reason was proposed for the first time.

**Figure 8:**
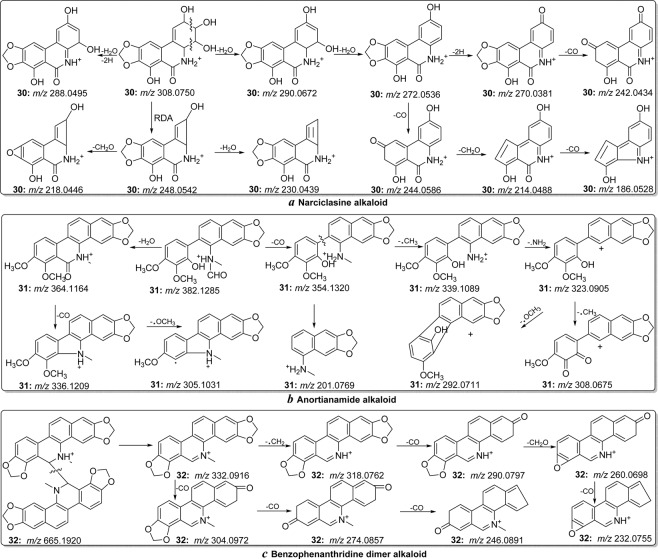
The proposed fragmentation behaviours of narciclasine (**a**), anortianamide (**b**) and benzophenanthridine dimer (**c**).

From the MS/MS spectra of narciclasine, anortianamide and benzophenanthridine dimer, some lower *m/z* region fragment ions (compared with the protonated molecules) were generated mainly by the cleavage of the alkaloid skeleton. The characteristic fragmentation behaviour played an important role in distinguishing dihydrobenzophenanthridine, 7,8-dihydroprotoberberine, benzophenanthridine, protoberberine, benzoquinoline and tetrahydrobenzoquinoline-type alkaloids^14,15,24^. In the MS/MS spectrum of narciclasine (**30**) (Fig. S1), the ions at *m/z* 290.0672 and 272.0536 were formed, corresponding to successively neutral loss of H_2_O molecules from the protonated ion at *m/z* 308.0750 (Fig. 8(a))^24^. However, in the MS/MS spectrum of anortianamide (**31**) (Fig. S1), only neutral loss of a H_2_O molecule from the ion *m/z* 382.1285 and a fragment ion at *m/z* 364.1164 were observed (Fig. 8(b)). In addition, the predominant ion at *m/z* 248.0542 was observed in the mass spectrum of alkaloid **30** corresponding to the RDA reaction. The benzophenanthridine dimer alkaloid could be easily categorized by the presence of [1/2(M-1.0078)]^+^ ions (Fig. 8(c)). These characteristic fragmentation behaviours and fragments played an important role in the discrimination of narciclasine, anortianamide and benzophenanthridine dimer-types alkaloids. The proposed flowchart for identification of these three alkaloid types is shown in Fig. 4. The proposed fragmentation behaviours of these alkaloids are shown in Fig. 8^14,15,24^.

**Figure 9:**
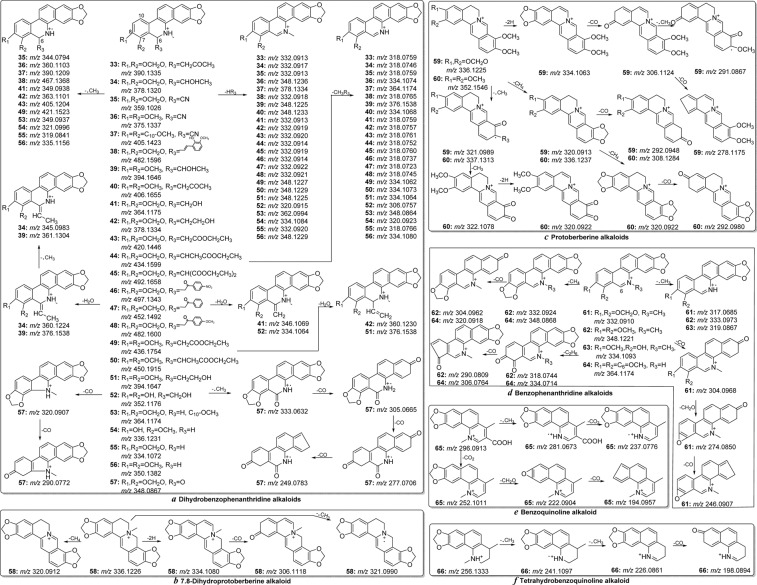
The proposed fragmentation behaviours of dihydrobenzophenanthridine (**a**), 7,8-dihydroprotoberberine (**b**), protoberberine (**c**), benzophenanthridine (**d**), benzoquinoline (**e**) and tetrahydrobenzoquinoline (**f**).

The MS/MS spectra of dihydrobenzophenanthridine, 7,8-dihydroprotoberberine, benzophenanthridine, protoberberine, benzoquinoline and tetrahydrobenzoquinoline(Fig. S1) are different from those of other alkaloid types. The fragments were formed mainly by the loss of some substituent groups because of the conjugated structure. The [M-HR_3_]^+^ and [M-CH_3_R_3_]^+^ fragment ions were observed for the dihydrobenzophenanthridine-type alkaloids corresponding to loss of the substituent at C-6 (R_3_) with vicinal groups (H and CH_3_)^14,15,21^. Taking alkaloid **33** as an example, the fragments at *m/z* 332.0913 (−1.20 ppm) and 318.0759 (−0.62 ppm) were simultaneously formed by the loss of CH_3_COCH_3_ ([M-HR_3_]^+^) and CH_3_CH_2_COCH_3_ ([M-CH_3_R_3_]^+^) moieties, respectively, from the protonated molecule at *m/z* 390.1335 (−0.25 ppm) (Fig. 9(a))^15^. The loss of *N*-methyl and vicinal H-atom from the ion at *m/z* 336.1226 and the formation of [M-CH_4_]^+^ fragments (*m/z* 320.0912) were detected for 7,8-dihydroprotoberberine (**58**), which were regarded as characteristic fragmentation behaviours of 7,8-dihydroprotoberberine-type alkaloids(Fig. 9(b)). The [fragment ion-2H]^+^ ions (*e.g*, *m/z* 334.1063 and 320.1266 for alkaloids **59** and **60**, respectively) were detected for protoberberine-type alkaloids, which corresponded to a loss of the H-atoms at C-5 and C-6, forming the conjugated system^8,15^ (Fig. 9(c)). However, the characteristic ion was also observed for benzophenanthridine, which always appeared along with the loss of a CO moiety and formation of [fragment ion-CH_2_O]^+^ ions (*e.g*, *m/z* 274.0850 and 302.0809 for alkaloids **61** and **62**, respectively) (Fig. 9(d))^15^. In the MS/MS spectrum of alkaloid **65** (benzoquinoline alkaloid) (Fig. S1), the [fragment ion-CO_2_]^+^ ions (*m/z* 252.1011 and 237.0776) were detected, corresponding to the existence of the -COOH group (Fig. 9(e))^28^. The six types of alkaloids could be characterized by their special fragmentation behaviours and fragment ions (the fragmentation pathway of tetrahydrobenzoquinoline alkaloid (**66**) is provided in Fig. 9(f)). The proposed flowchart for identification of each alkaloid type is shown in Fig. 4. The proposed fragmentation behaviours of these alkaloids are shown in Fig. 9^8,14,15,21,29^.

In addition to the above characteristic fragmentation patterns, some common fragmentation behaviours were also observed for these types of isoquinoline alkaloids. Alkaloids with vicinal methoxy and hydroxy, vicinal methoxy and methylenedioxy groups could form the [M-CH_3_OH]^+^, [M-CH_4_]^+^ and [fragment ion-CH_2_O]^+^ or [fragment ion-CO]^+^ fragment ions, respectively. These characteristic pathways have been discussed. In addition, alkaloids with methoxy groups could produce [fragment ion-CH_3_]^+^ fragments by loss of a CH_3_ radical, and continual neutral ejection of a CO molecule and formation [fragment ion-CH_3_-CO]^+^ ions were also observed^15^. In the MS/MS spectra of alkaloids **59** and **60** (Fig. S1), the ions at *m/z* 291.0867 and 322.1078 were formed by the loss of a CH_3_ radical from the precursor ions at *m/z* 306.1124 and 337.1313, respectively. Subsequently, continual ejection of a CO molecule led to the formation of fragments at *m/z* 263.0809 and 294.1128^8^. Alkaloid with quaternary *N*-methyl group could form the [M-CH_3_]^+^ fragment ion. In the MS/MS spectra of alkaloids **61** and **65** (Fig. S1), the fragments at *m/z* 317.0685 and 281.0673 were presented by the loss of a CH_3_ radical from quaternary *N*-centre^15^.

**Figure 10:**
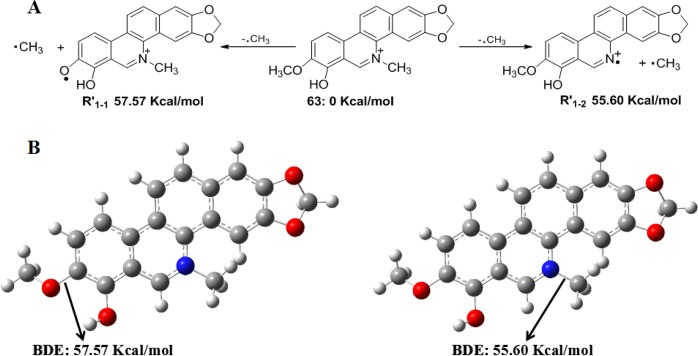
Two theoretical calculation methods (**A**,**B**) for the loss of a CH_3_ radical from alkaloid **63**.

Some interesting common fragmentation pathways draw our attention. Taking alkaloid **63** as an example, the loss of a CH_3_ radical from the mother ion at *m/z* 334.1093 could give the high-abundance fragment ion at *m/z* 319.0867. Normally, among *N*-CH_3_ and O-CH_3_, it was difficult to demonstrate which one was favoured regarding the loss of the CH_3_ moiety in the MS spectrum. Herein, two calculation methods were used to predict the more reasonable fragmentation pathway. Two hypothetical losses of the CH_3_ radical from methoxyl at C-8 or *N*-methyl in the mother ion would give **R’**_**1-1**_ and **R’**_**1-2**_ fragmentation, respectively (Fig. 10(A)). The relative energy of **R’**_**1-2**_ was 55.60 kcal/mol, which was lower than that of **R’**_**1-1**_, indicated that **R’**_**1-2**_ was more stable to give high-abundance fragment ions. In addition to the above method, the bond dissociation energies of *N*-CH_3_ and O-CH_3_ were calculated (Fig. 10(B)). The dissociation energy of *N*-CH_3_ was 55.60 kcal/mol, which was 1.97 kcal/mol lower than that of O-CH_3_. This result suggested that the bond dissociation between the *N*-atom and CH_3_ was favoured. The calculation results indicated that the mother ion at *m/z* 334.1093 tends to lose the CH_3_ moiety from the *N*-atom and subsequently gave the high-abundance molecular ion peak at *m/z* 319.0867.

## Summary

In this study, the MS/MS fragmentation behaviours of 66 compounds, which belong to 19 types of isoquinoline alkaloids, were investigated on a Q-TOF/MS spectrometer in ESI^+^ mode. These isoquinoline alkaloids could be divided into three categories primarily by the characteristic [M-NHR_1_R_2_]^+^ fragment ion. Type I and II alkaloids (compounds **1**–**29**) could form high- and low- abundance [M-NHR_1_R_2_]^+^ ions, respectively. However, the characteristic fragments were not generated for type III alkaloids (compounds **30**–**66**) because of the existence of a *p*-π conjugated system in its structure. Furthermore, each type of alkaloid was classified by its characteristic MS/MS fragmentation patterns and fragment ions (Fig. 4). The high-abundance [M-NHR_1_R_2_]^+^ fragments of benzyltetrahydroisoquinoline and aporphine-type alkaloid have been reported in some previous studies but rarely mentioned for ipecac, chelidonine, and bisbenzyltetrahydroisoquinoline-type alkaloids (type I)^15,16^. The low-abundance [M-NHR_1_R_2_]^+^ ions of type II alkaloids were observed and reported for the first time. The mechanism of formation of [M-NHR_1_R_2_]^+^ fragment ions was also investigated by computational chemistry. The reason for the absence of characteristic [M-NHR_1_R_2_]^+^ fragment ions of type III alkaloids was proposed. In addition, five common fragmentation pathways were observed and summarized for isoquinoline alkaloids: (1) Alkaloids with vicinal methoxy and hydroxy groups could produce the characteristic fragments by neutral loss of a CH_3_OH molecule. (2) Alkaloids with vicinal methoxy groups could form the characteristic ions by eliminating of a CH_4_ moiety. (3) Alkaloids with methylenedioxy groups could generate interesting fragments by loss of a CH_2_O moiety or CO molecule. (4) Alkaloids with methoxy groups could produce distinctive ions by ejection of a methyl radical and CO molecule. (5) Alkaloids with quaternary *N*-methyl groups could form the [M-CH_3_]^+^ fragment ion^10,15^. These characteristic fragmentation pathways of isoquinoline alkaloids provided a solid foundation for the structural elucidation of similar metabolites in plant-derived medicines.

## Experiment

### Materials and reagents

HPLC-grade formic acid and acetonitrile were purchased from Merck (Darmstadt, Germany) and ROE (Newark, New Castle, USA), respectively. Deionized water was purified using a Milli-Q system (MA, USA). The 22 references, 3′-hydroxy-*N*-methylcoclaurine (**3**), reticuline (**4**), norlaudanosoline (**5**), 6-*O*-methylnorlaudanosoline (**6**), norreticuline (**7**), magnoflorine (**8**), isocorydine (**9**), cephaeline (**10**), emetine (**11**), chelidonine (**12**), tetrandrin (**13**), stylopine (**16**), tetrahydrocolumbamine (**17**), canadine (**18**), tetrahydropalmatine (**19**), *N*-methylcheilanthifoline (**21**), phellodendrine (**22**), morphine (**27**), codeine (**28**), noscapine (**29**), narciclasine (**30**), and palmatine chloride (**60**), were purchased from the National Institutes for Food and Drug Control (Beijing, China). The 29 reference alkaloids, namely, *N*-methylscoulerine (**24**), protopine (**25**), allocryprotopine (**26**), anortianamide (**31**), sanguinarine dimer (**32**), 6-acetonyldihydrosanguinarine (**33**), (1′ → 6)-hydroxy- ethyldihydrosanguinarine (**34**), 6-cyanodihydrosanguinarine (**35**), 6-cyanodihydrochelerythrine (**36**), 6-cyanodihydrochelilutine (**37**), maclekarpine E (**38**), (1′ → 6)-hydroxyethyldihydrochelerythrine (**39**), 6-acetonyldihydrochelerythrine (**40**), 6-hydroxymethylsanguinarine (**41**), 6-hydroxyethyldihydro- chelerythrine (**51**), 6-hydroxymethyl-7,8-demethylenedihydrochelerythrine (**52**), dihydrochelirubine (**53**), 8-demethyldihydrochelerythrine (**54**), dihydrosanguinarine (**55**), dihydrochelerythrine (**56**), oxysanguinarine (**57**), 7,8-dihydrocoptisine (**58**), berberine (**59**), sanguinarine (**61**), chelerythrine (**62**), 7-demethylchelerythrine (**63**), 6-methoxy-norchelerythrine (**64**), 2,3-methylenedioxy-7,10-dimethyl- 8-carboxyl-benzoquinoline (**65**), and 2,3-methylenedioxy-7,10-dimethyl-7,8,9,10-tetrahydro- benzoquinoline (**66**), were separated and identified from the plant-derived medicine named *Macleaya cordata* by our laboratory. The remaining 15 references, namely, coclaurine (**1**), *N*-methylcoclaurine (**2**), scoulerine (**14**), cheilanthifoline (**15**), *N*-methylstylopine (**20**), *N*-methyltetrahydroberberine (**23**), 6-ethoxy-dihydrosanguinarine (**42**), ethyl 2′-(dihydrosanguinarine-6-yl)acetate (**43**), ethyl 2′-(dihydro- sanguinarine-6-yl)propanoate(**44**), ethyl 2′-(dihydrosanguinarine-6-yl)maonlate (**45**), 1′-(4-nitrophenyl)−2′-(dihydro-sanguinarine-6-yl)ethanone(**46**), 1′-phenyl-2′-(dihydrosanguinarine–6-yl)ethanone(**47**), 1′-(4-methoxyphenyl)-2′-(dihydro-sanguinarine-6-yl)ethanone (**48**), ethyl 2′-(dihydrochelerythrine-6-yl)acetate (**49**), and ethyl 2′-(dihydrochelerythrine-6-yl)propanoate (**50**), were synthesized in our laboratory. The above 66 references (Table S1) were used to investigate the fragmentation behaviours of 19 types of isoquinoline alkaloids.

### Reference preparation and analysis

Approximately 2.0 mg alkaloid was dissolved in 10 mL methanol by an ultrasonic bath for 5 min. A portion of the methanol solution was filtered through a 0.22 μm nylon membrane. A 2 μL sample was injected into Q-TOF/MS directly by an Agilent 1290 high-performance liquid chromatography (HPLC) system (Agilient Technologies, USA) consisting of an auto-sampler, a rapid resolution binary pump, a vacuum degasser, a thermostatted column compartment and a tunable UV detector. However, the chromatographic column was not equipped with the HPLC system for rapidly obtaining the MS and MS/MS data of references.

### Q-TOF/MS conditions

Mass spectra were acquired using a 6530 Q-TOF/MS accurate-mass spectrometer (Agilent Technologies, Palo Alto, CA, USA) equipped with an ESI source in positive ion mode. The conditions of the Q-TOF-MS were optimized as follows: sheath gas temperature: 350 °C; gas temperature: 300 °C; nebulizer pressure: 35 psi; sheath gas flow: 11 L/min; drying gas: 8 L/min; fragmentor voltage: 175 V; skimmer voltage: 65 V; capillary voltage: 3500 V. The TOF mass spectrometry was calibrated in ESI^+^ mode before sample analysis using reference masses at *m/z* 121.0508 and 922.0097 to obtain high-accuracy mass measurements. The MS/MS experiments were performed using variable collision energy (10–50 eV), which was optimized for each individual reference.

### Theoretical calculations

All theoretical calculations for geometries of the neutral and protonated molecules, as well as the product ions, were performed by using the B3LYP function in combination with the 6–31 G(*d*) basis set in the Gaussian 03 package of programs. An unrestricted open-spell calculation (UB3LYP) was used for odd electron ions. The candidate structures of protonated molecules, fragment ions, neutral molecules and transition states were optimized by calculating the force constants, while no symmetry constraints were imposed in the process of optimization^29,30^. All optimized structures were subjected to vibrational frequency analysis for zero-point energy correction^29,31^. The energies of each optimized structure are the sum of electronic and thermal energies. The optimized structures were visualized by Gauss View (version 3.09).

## Supplementary information

Supplementary Information.
