# Supplementary material for: Investigation of fragmentation behaviours of isoquinoline alkaloids by mass spectrometry combined with computational chemistry
Source: Sci Rep. 2020 Jan 20;10:733. doi: 10.1038/s41598-019-57406-7 (PMC6970995; doi:10.1038/s41598-019-57406-7)

**Investigation of fragmentation behaviours of isoquinoline alkaloids by**

**mass spectrometry combined with computational chemistry**

Zhixing Qing^1,2^**^†^**, Yuqin Xu^4^**^†^**, Liuyi Yu^3^, Jinghong Liu^1^, Xiuqiong Huang^1^, Zhaoshan Tang^5^, Pi Cheng^1*^, Jianguo Zeng^1,5*^

1. Hunan Key Laboratory of Traditional Chinese Veterinary Medicine, Hunan Agricultural University, Changsha, 410128, China.
2. College of Food Science and Technology, Hunan Agricultural University, Changsha, 410128, China.

3. School of pharmacy, Hunan University of Chinese Medicine, Changsha 410208, China.

4. Department of pharmacy, First Affiliated Hospital of Hunan University of Chinese Medicine, Changsha, 410007, China.

5. Micolta Bioresource Inc., Changsha 410005, China.

**^†^:** *These authors contribution equally to this work*.

Table S1 The specific information of 66 references

| NO. | Name | Formula | [M+H]^+^ or [M]^+^ (*m/z*) | Error*^e^*  (ppm) | Characteristic MS/MS fragment ions (*m/z*) | Source | Supplier | Purity |
| --- | --- | --- | --- | --- | --- | --- | --- | --- |
| 1 | Coclaurine | C_17_H_19_NO_3_ | 286.1445 | 2.4 | 269.1180(2.6)*^d^*, 237.0899(-5.0), 209.0967(2.3), 178.0849(-7.8), 175.0755(-2.2), 143.0490(-0.6), 121.0661(-5.7), 107.0488(-2.8) | S*^a^* | / | 96% |
| 2 | *N*-methylcoclaurine | C_18_H_21_NO_3_ | 300.1582 | -4.0 | 269.1169(-1.1), 237.0913(1.2), 209.0960(-0.4), 192.1012(-3.6), 175.0750(-2.2), 143.0482(-6.9), 121.0642(-4.9), 107.0488(-2.8) | S | / | 96% |
| 3 | 3’-hydroxy-*N*-  methylcoclaurine | C_18_H_21_NO_4_ | 316.1545 | 0.6 | 285.1137(5.6), 253.0866(2.7), 225.0912(0.8), 192.1018(-0.5), 175.0749(-2.8), 143.0486(-4.1), 137.0589(-5.8), 123.0434(-5.6) | P*^b^* | TRC, Canada | 98% |
| 4 | Reticuline | C_19_H_23_NO_4_ | 330.1714 | 4.2 | 229.1279(0.3), 267.1011(-1.8), 239.1053(-5.8), 192.1020(0.5), 175.0753(-0.5), 143.0485(-4.8), 151.0754(0.0), 137.0592(-3.6) | P | Biobiopha, China | 98% |
| 5 | Norlaudanosoline | C_16_H_17_NO_4_ | 288.1233 | 1.0 | 271.0984(7.0), 253.0875(6.3), 225.0917(-1.3), 164.0709(-7.8), 161.0604(-4.3), 143.0489(-2.0),  137.0591(-4.3), 123.0440(-0.8) | P | TRC, Canada | 98% |
| 6 | 1. *O*-methyl- 2. norlaudanosoline | C_17_H_20_NO_4_ | 302.1394 | 2.3 | 285.1134(4.5), 253.0860(0.3), 225.0910(-0.8), 178.0866(1.6), 175.0756(1.1), 143.0499(4.8),  137.0598(0.7), 123.0445(3.2) | P | TRC, Canada | 96% |
| 7 | Norreticuline | C_18_H_21_NO_4_ | 316.1552 | 2.8 | 299.1281(1.0), 267.0993(-8.6), 239.1063(-1.6), 178.0861(-7.8), 175.0749(-2.8), 151.0753(-0.6),  143.0486(-4.1), 137.0593(-2.9) | P | Specs, USA | 98% |
| 8 | Magnoflorine | C_20_H_24_NO_4_ | 342.1701 | 0.2 | 297.1114(-2.3), 282.0895(3.5), 279.1040(8.9), 265.0856(-1.1), 175.0749(6.3) | P | NIFDC, China | 98% |
| 9 | Isocorydine | C_20_H_24_NO_4_ | 342.1696 | -1.1 | 311.1279(0.3), 296.1035(-2.3), 279.1014(-0.7), 264.0780(-2.2), 251.1053(-5.5), 236.0826(-4.6),  248.0829(-3.2), 221.0590(-5.8) | P | NIFDC, China | 98% |
| 10 | Cephaeline | C_28_H_38_N_2_O_4_ | 467.2898 | -2.5 | 450.2633(-2.6), 434.2325(-1.6),  422.2318(-3.3), 288.1948(-3.4),  272.1639(-2.2), 260.1634(-4.2),  246.1485(1.6), 206.1164(-5.8) | P | TRC, Canada | 98% |
| 11 | Emetine | C_29_H_39_N_2_O_4_ | 481.3063 | 0.4 | 464.2797(0.2), 448.2486(0.6), 436.2492(2.0), 288.1960(0.6),  274.1809(2.5), 272.1648(1.1), 246.1494(-2.0), 206.1174(-0.9) | P | TRC, Canada | 98% |
| 12 | Chelidonine | C_20_H_19_NO_5_ | 354.1313 | -6.4 | 336.1210(-5.9), 323.0893(-6.5), 305.0785(-7.5), 295.0941(-8.1),  275.0683(-7.2), 247.0734(-8.0), 163.0381(-5.5), 135.0426(11.1) | P | Sigma, USA | 98% |
| 13 | Tetrandrin | C_38_H_42_N_2_O_6_ | 623.3121 | 0.0 | 580.2694(0.0), 607.2807(0.6), 592.2707(2.1), 396.2042(-0.5),  381.1807(-0.5), 350.1396(2.5), 174.0902(-6.3), 162.0901(-7.4) | P | NIFDC, China | 98% |
| 14 | Scoulerine | C_19_H_21_NO_4_ | 328.1542 | -0.3 | 313.1303(-1.92),311.1273(-1.6),  296.1268(-4.4), 151.0751(2.0), 178.0859(-2.2), 176.0696(-5.6), | S | / | 96% |
| 15 | Cheilanthifoline | C_19_H_19_NO_4_ | 326.1381 | -1.8 | 309.1121(0.0), 294.1097(-9.5), 311.1098(#), 149.0603(4.0), 178.0851(-6.7), 176.0687(#) | S | / | 94% |
| 16 | Stylopine | C_19_H_17_NO_4_ | 324.1226 | -1.2 | 307.0965(0.0), 149.0594(-2.0),  176.0704(-1.1), 174.0543(-4.0) | P | Chromadex, USA | 98.9% |
| 17 | Tetrahydro-  columbamine | C_20_H_23_NO_4_ | 342.1685 | -4.3 | 325.1467(0.8), 293.1178(0.2),  327.1472(2.1),165.0904(-0.6), 178.0854(-5.0), 176.0691(-8.5), | P | Biobiopha, China | 98% |
| 18 | Canadine | C_20_H_21_NO_4_ | 340.1543 | 0.0 | 323.1290(3.7), 165.0908(1.8),  176.0705(-0.5), 174.0550(0.0) | P | TRC, Canada | 98% |
| 19 | Tetrahydro-  palmatine | C_21_H_25_NO_4_ | 356.1865 | 2.5 | 339.1601(2.9), 165.0920(9.1),  192.1030(8.3), 190.0877(7.3),  177.0797(7.3) | P | NIFDC, China | 98% |
| 20 | *N*-methylstylopine | C_20_H_20_NO_4_ | 338.1392 | 1.1 | 307.0953(-4.2), 322.1091(5.2) ,  149.0591(-7.3), 190.0860(-1.5),  188.0703(-1.5), 149.0591(-7.3),  190.0860(-1.5) | S | / | 94% |
| 21 | *N*-methyl-  cheilanthifoline | C_19_H_17_NO_4_ | 340.1543 | -0.8 | 309.1121 (0.0), 324.1231(0.3) ,  192.1018(-0.5),190.0841(#), 151.0744(-9.9), 177.0792(3.9) | P | NIFDC, China | 98% |
| 22 | Phellodendrine | C_20_H_23_NO_4_ | 342.1700 | 0.0 | 311.1284(1.9),192.1017(-0.5), 190.0859(-2.1), 177.0778(-3.9) | P | NIFDC, China | 98% |
| 23 | *N*-methyltetrahydro-berberine | C_21_H_23_NO_4_ | 354.1705 | 1.4 | 338.1403(4.7), 323.1287(2.7) ,  190.0872(4.7),188.0714(4.2),  149.0606(2.6),165.0914(-0.6) | S | / | 94% |
| 24 | *N*-methylscoulerine | C_20_H_23_NO_4_ | 342.1709 | 2.6 | 326.1378(-2.7), 311.1280(0.6),  192.1025(3.1),190.0863(0.0), 177.0787(1.1),151.0751(-5.3) | I*^c^* | / | 96% |
| 25 | Protopine | C_20_H_19_NO_5_ | 354.1323 | -3.6 | 354.1323(-3.6), 323.0898(-4.9),  206.0813(0.4),189.0772(-6.8),  149.0591(-4.0) | I | / | 98% |
| 26 | Allocryprotopine | C_21_H_23_NO_5_ | 370.1641 | -2.1 | 339.1220 (-2.0), 165.0912(-1.8), 206.0813(0.4), 189.0775(-5.7) | I | / | 98% |
| 27 | Morphine | C_17_H_19_NO_3_ | 286.1432 | -2.1 | 268.1323(-3.3), 229.0857(-0.8),  255.101(-2.3), 227.0716(5.7), 211.0748(-2.8), 201.090(-4.9), 193.0685(-2.5), 183.0797(-3.8),  165.0697(-1.2), 58.0659(#), | P | Cerilliant, USA | 1±0.005  mg/mL |
| 28 | Codeine | C_17_H_19_NO_3_ | 300.1598 | 1.3 | 282.1486(-1.0), 269.1306(-9.7)  241.0844(6.2), 225.0921(-4.8), 193.0729(#), 165.0615(#),  58.062(#) | P | Cerilliant, USA | 1±0.005  mg/mL |
| 29 | Noscapine | C_22_H_23_NO_7_ | 414.1548 | 0.2 | 396.143(-3.3), 383.1143(-4.7),  381.1205(0.5), 353.1018(-0.5),  323.0906(-2.4), 220.096(3.6), 205.0713(9.7), 179.0693(5.5),  164.0828(#) | P | Sigma, USA | 98% |
| 30 | Narciclasine | C_14_H_13_NO_7_ | 308.0750 | -6.4 | 290.0672(4.4), 288.0495(-2.7),  272.0536(-6.2), 270.0381(-5.9),  248.0542(-4.4), 244.0586(-7.3),  242.0434(-5.7), 230.0439(-3.9),  218.0446(-0.9), 214.0488(-5.1) | P | Sigma, USA | 98% |
| 31 | Anortianamide | C_21_H_19_NO_6_ | 382.1285 | 0.0 | 364.1164(-4.1), 354.1320(-4.5),  339.1089(-3.5), 336.1209(-6.2),  323.0905(-2.8), 308.0675(-1.3),  305.1031(-4.9), 292.0711(-6.5),  201.0769(-7.4) | I | / | 94% |
| 32 | Sanguinarine dimer | C_40_H_28_N_2_O_8_ | 665.1920 | 0.3 | 332.0916(-0.3),318.0762(0.3),  304.0972(1.3), 290.0797(-5.1),  274.0857(-2.1), 246.0891(-8.9),  260.0698(-3.0), 232.0755(-0.8) | I | / | 98% |
| 33 | 1. Acetonyldihydro   -sanguinarine | C_23_H_19_NO_5_ | 390.1335 | -0.2 | 332.0913(-1.2), 318.0759(-0.6) | I | / | > 95% |
| 34 | (1’→6)hydroxy-  Ethyldihydro-  sanguinarine | C_22_H_19_NO_5_ | 378.1320 | -4.2 | 332.0917(0.0), 318.0746(-4.7),  345.0983(-3.7), 360.1224(-1.6) | I | / | > 95% |
| 35 | 6-cyanodihydrosanguinarine | C_21_H_14_N_2_O_4_ | 359.1026 | 0.0 | 332.0913(-1.2), 318.0759(-0.6),  344.0794(0.5) | I | / | 98% |
| 36 | 1. Cyanodihydro-   chelerythrine | C_22_H_18_N_2_O_4_ | 375.1337 | -0.5 | 390.1336(-0.2), 360.1103(-0.5),  348.1236(1.7), 334.1074(0.0) | I | / | 98% |
| 37 | 1. Cyanodihydro-   chelilutine | C_23_H_20_N_2_O_5_ | 405.1423 | -5.43 | 390.1209(0.2), 378.1334(-0.5),  364.1174(-1.3) | I | / | 94% |
| 38 | Maclekarpine E | C_29_H_23_NO_6_ | 482.1596 | -0.41 | 467.1368(1.0), 332.0918(0.3), 318.0765(1.2) | I | / | > 95% |
| 39 | (1’→6)-hydroxyethyl-dihydro-  chelerythrine | C_23_H_23_NO_5_ | 394.1646 | -0.76 | 376.1538(-1.3), 361.1304(-1.3), 348.1225(-1.4) | I | / | > 95% |
| 40 | 1. Acetonyldihydro   -chelerythrine | C_24_H_23_NO_5_ | 406.1655 | 1.47 | 348.1233(0.8), 334.1068(-1.7) | I | / | > 95% |
| 41 | 1. Hydroxymethyl   -sanguinarine | C_21_H_17_NO_5_ | 364.1175 | -1.0 | 349.0938(-2.0), 346.1069(-1.4), 332.0913(-1.2), 318.0759(-0.6) | I | / | > 95% |
| 42 | 1. ethoxy-dihydro-   sanguinarine | C_22_H_19_NO_5_ | 378.1334 | -0.52 | 363.1101(0.0), 360.1230(0.0), 332.0919(0.6), 318.0757(-1.2) | S | / | > 95% |
| 43 | ethyl 2’-(dihydrosanguinarine-6-yl)acetate | C_24_H_21_NO_6_ | 420.1446 | 0.95 | 405.1204(-0.7), 332.0920(0.9), 318.0761(0.0) | S | / | > 95% |
| 44 | ethyl 2’-(dihydro  sanguinarine- 6-yl)propanoate | C_25_H_23_NO_6_ | 434.1599 | 0.23 | 332.0914(-0.9), 318.0761(-2.8) | S | / | > 95% |
| 45 | ethyl 2’-(dihydrosanguinarine-6-yl)maonlate | C_27_H_25_NO_8_ | 492.1658 | 1.01 | 332.0919(0.6), 318.0760(-0.3) | S | / | > 95% |
| 46 | 1’-(4-nitrophenyl)-  2’-(dihydro -sanguinarine-6-yl)ethanone | C_28_H_20_N_2_O_7_ | 497.1343 | 0 | 332.0914(-0.9), 318.0737(-7.5) | S | / | > 95% |
| 47 | 1’-phenyl-2’-(dihydrosanguinarine-  6-yl)ethanone | C_28_H_21_NO_5_ | 452.1492 | 0 | 332.0922(1.5), 318.0723(#) | S | / | > 95% |
| 48 | 1’-(4-methoxyphenyl)-2’-(dihydro-  sanguinarine-6-yl)ethanone | C_29_H_23_NO_6_ | 482.1600 | 0.41 | 482.1600(0.4), 332.0921(1.2), 318.0745(-5.0) | S | / | > 95% |
| 49 | ethyl 2’-(dihydrochelerythrine-6-yl)acetate | C_25_H_25_NO_6_ | 436.1754 | 0.22 | 421.1523(0.7), 348.1227(-0.8), 334.1062(-3.5), 332.0921(1.2) | S | / | > 95% |
| 50 | ethyl 2’-(dihydrochelerythrine-6-yl)  propanoate | C_26_H_27_NO_6_ | 450.1915 | 0.88 | 348.1229(-0.2), 334.1073(-0.29) | S | / | > 95% |
| 51 | 6-hydroxyethyldihydrochelerythrine | C_23_H_23_NO_5_ | 394.1647 | 0.50 | 376.1538(-1.3), 348.1225(-1.4), 334.1064(-2.9) | I | / | > 95% |
| 52 | 1. hydroxymethyl-7,8-demethy-   lenedihydrochelerythrine | C_20_H_17_NO_5_ | 352.1176 | 0.85 | 334.1064(2.9), 329.0915(-0.6), 306.0757(-1.3) | I | / | > 95% |
| 53 | dihydrochelirubine | C_21_H_17_NO_5_ | 364.1174 | -1.37 | 362.0994(-8.0), 349.0937(-2.2), 348.0864(-0.5) | I | / | > 95% |
| 54 | 8-demethyldihydrochelerythrine | C_20_H_17_NO_4_ | 336.1231 | 0.29 | 334.1084(2.9), 321.0996(0.0), 320.0923(1.8) | I | / | > 95% |
| 55 | Dihydro  sanguinarine | C_20_H_15_NO_4_ | 334.1072 | -0.59 | 332.0920(0.9), 319.0841(0.6),  318.0766(1.5) | I | / | > 95% |
| 56 | Dihydro  chelerythrine | C_21_H_19_NO_4_ | 350.1382 | 1.42 | 348.1229(-0.2), 335.1156(1.1), 334.1080(1.7) | I | / | > 95% |
| 57 | Oxysanguinarine | C_20_H_13_NO_5_ | 348.0867 | 0.28 | 333.0632(0.0), 320.0907(-3.1), 305.0665(-5.9), 277.0706(-9.7), 249.0783(0.4) | I | / | > 95% |
| 58 | 7,8-dihydro  coptisine | C_20_H_18_NO_4_ | 336.1226 | -1.1 | 334.108(1.7), 321.099 (-1.8), 320.0912(-1.5), 306.1118(-2.2) | I | / | > 95% |
| 59 | Berberine | C_20_H_18_NO_4_ | 336.1225 | -1.4 | 334.1063(0.0), 321.0989(2.1), 320.0913(-1.2), 306.1124(-0.3),  292.0948(-6.8), 278.1175(-0.3), | I | / | > 95% |
| 60 | Palmatine chloride | C_21_H_22_NO_4_ | 352.1546 | 1.4 | 337.1313(1.1), 336.1237(2.0), 322.1078(1.2), 320.0922(0.0),  308.1284(0.9), 292.098(4.1) | P | / | 98% |
| 61 | Sanguinarine | C_20_H_14_NO_4_^+^ | 332.0910 | -2.1 | 317.0685(0.6), 304.0968(0.0),  274.085(-4.7), 246.0907(-2.4) | I | / | > 95% |
| 62 | Chelerythrine | C_21_H_17_NO_4_^+^ | 348.1221 | -2.5 | 333.0973(-6.9), 332.0924(2.1), 318.0744(-5.3), 304.0962(-1.9),  290.0809(-1.0) | I | / | > 95% |
| 63 | 1. Demethyl 2. chelerythrine | C_20_H_16_NO_4_ | 334.1093 | 5.6 | 319.0867(8.7) | I | / | > 95% |
| 64 | 1. methoxy-   norchelerythrine | C_21_H_19_NO_5_ | 364.1174 | -1.3 | 348.0868(0.5), 334.0714(0.0), 320.0916(-0.3), 306.0764(0.9) | I | / | > 95% |
| 65 | 2,3-Methylenedioxy-7,10-dimethyl-8-  carboxyl-  benzoquinoline | C_16_H_17_NO_2_ | 296.0913 | -1.3 | 281.0673(-3.5), 237.0776(-3.7), 252.1011(-3.1), 222.0904(-4.0), 194.0957(-3.6) | I | / | > 95% |
| 66 | 2,3-Methylenedioxy-7,10-dimethyl-  7,8,9,10-tetrahydrobenzoquinoline | C_17_H_14_NO_4_ | 256.1333 | 0.3 | 241.1097(0.0), 226.0861(-0.8)  198.0894(-9.5) | I | / | > 95% |

*^a^* synthesized by our laboratory; *^b^* purchased from reagent company; *^c^* isolated by our laboratory; *^d^* error (ppm); *^e^* Error =(Measured *m/z* values－theoretical *m/z* values)/theoretical *m/z* values×10^6^ (ppm). ^#^ Error more than 10.0 ppm.

Figure S1 the MS/MS spectra of alkaloids **1**-**66**.


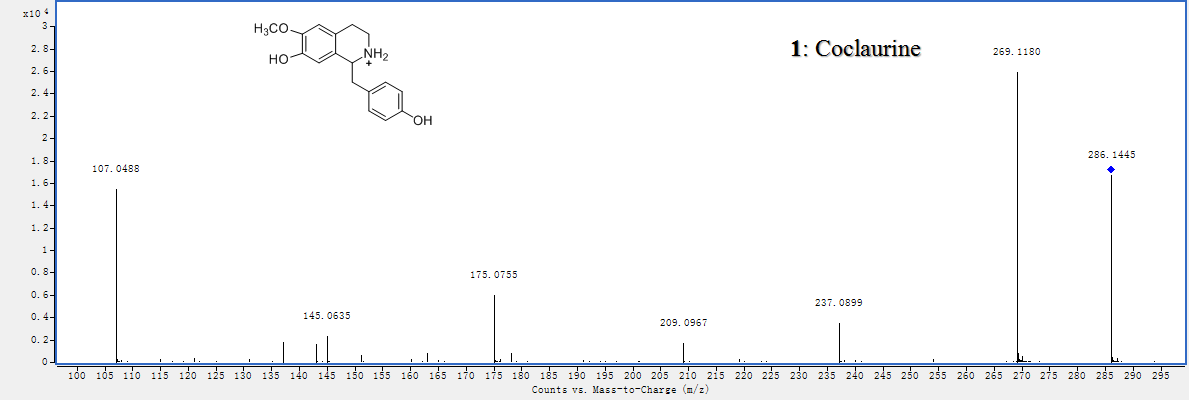


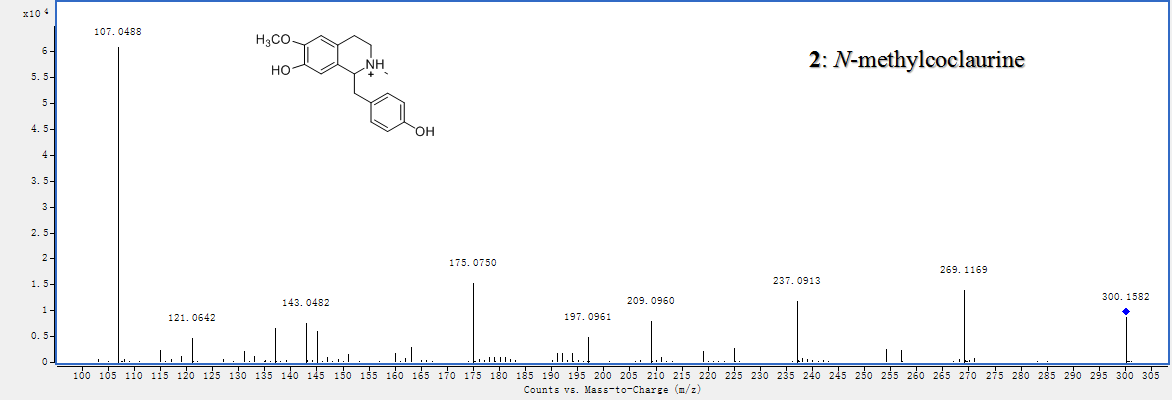


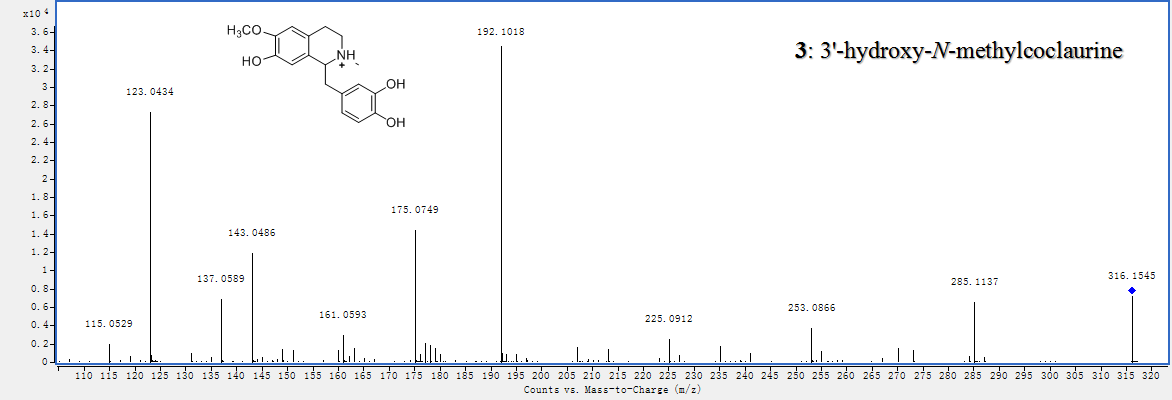


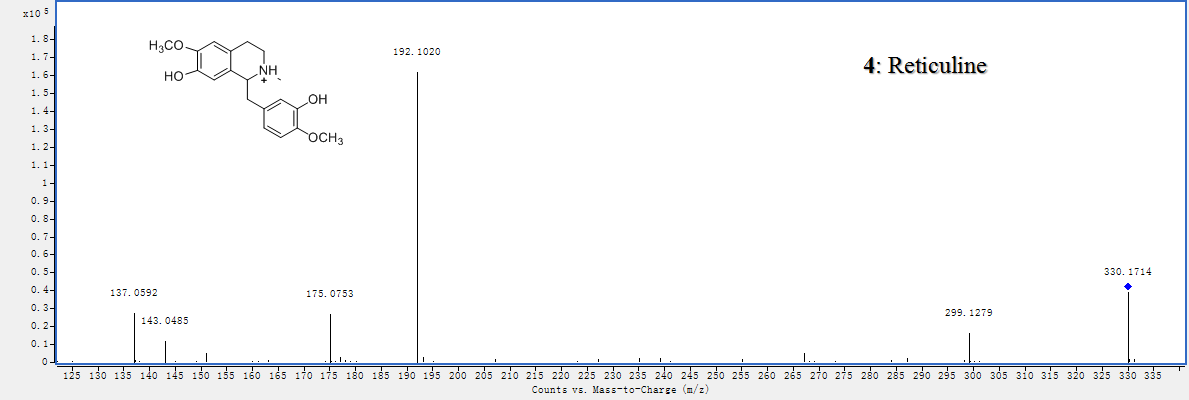


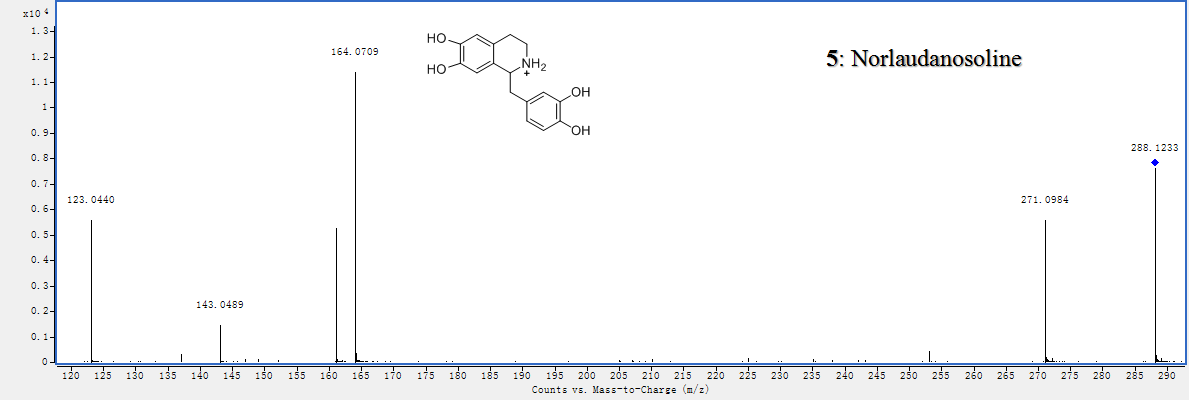


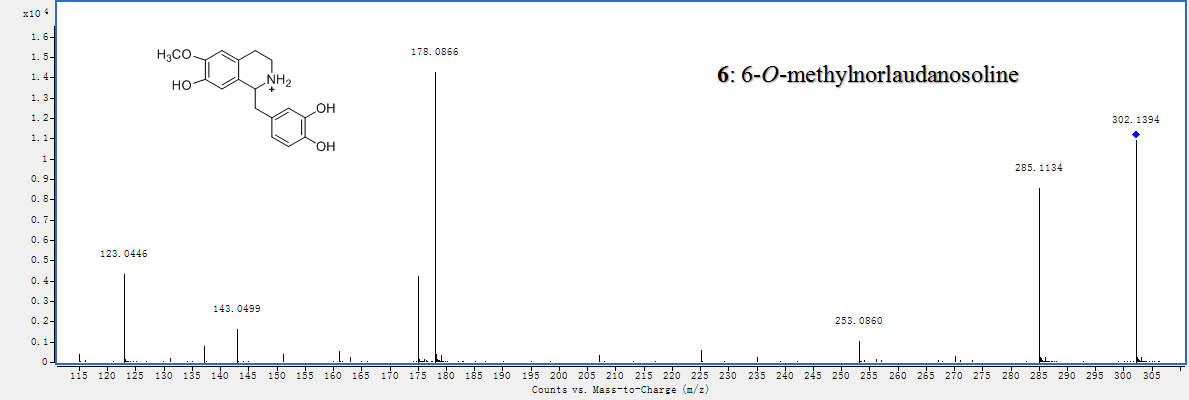


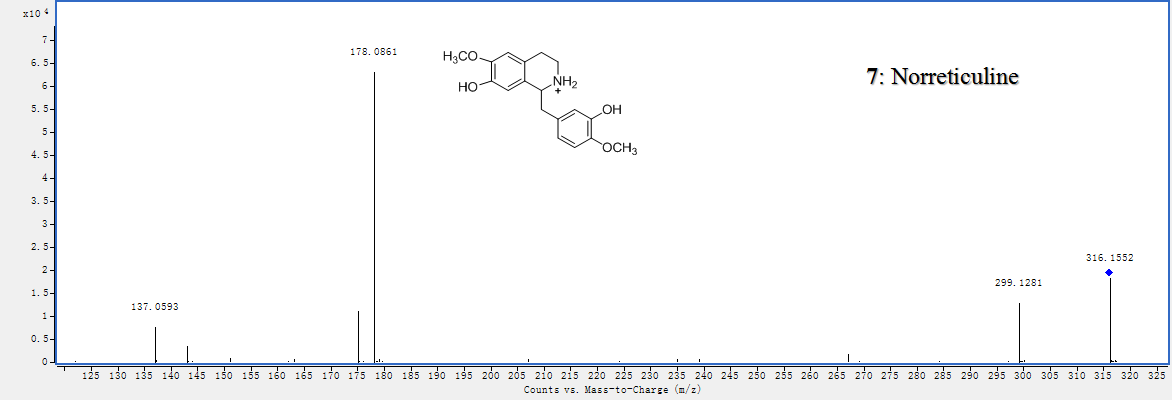


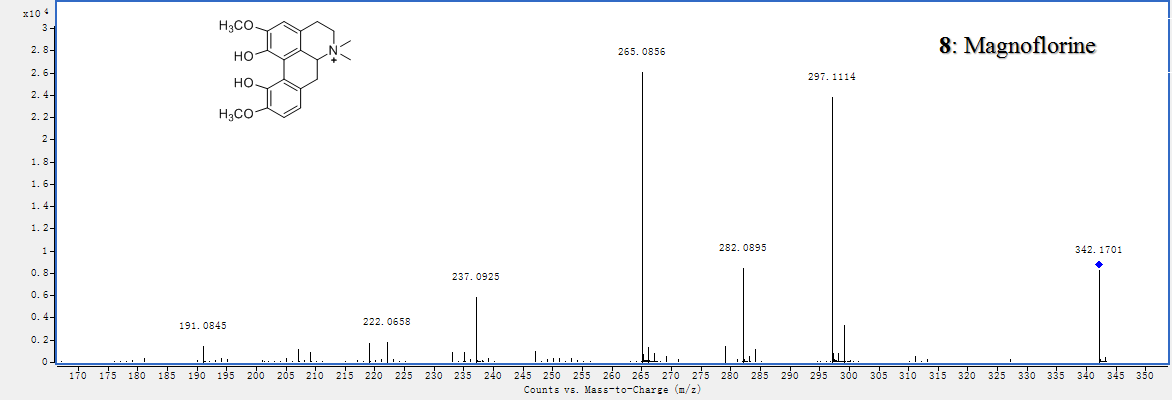


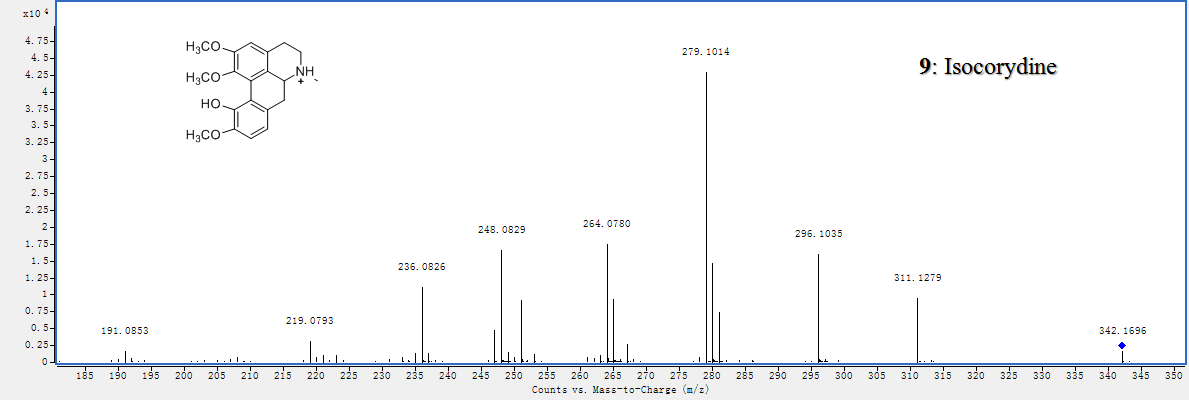


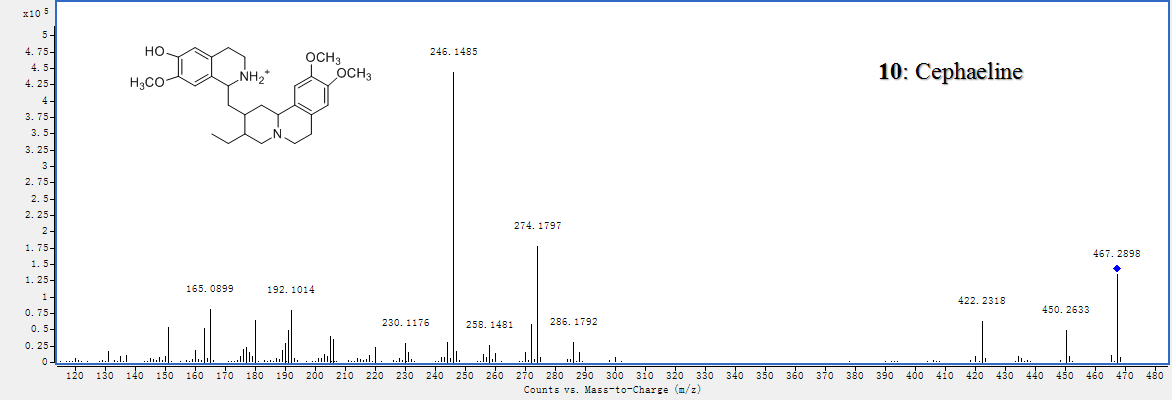


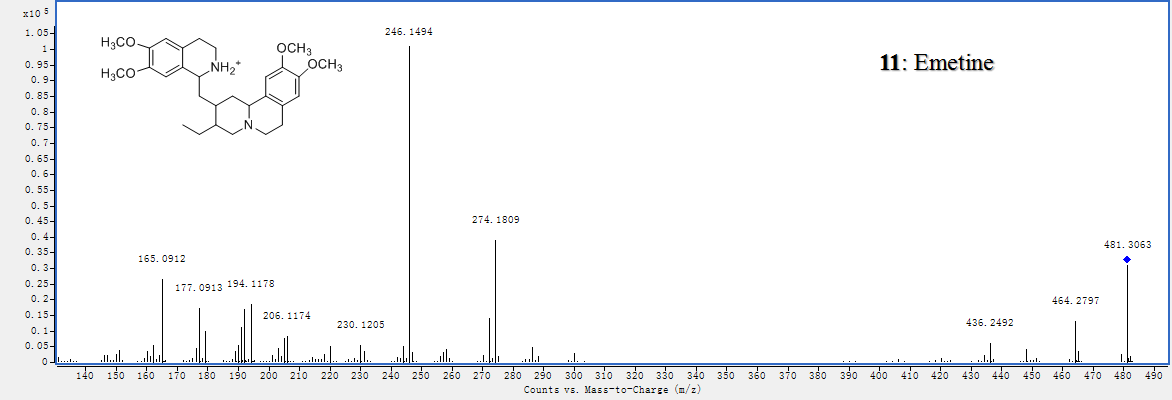


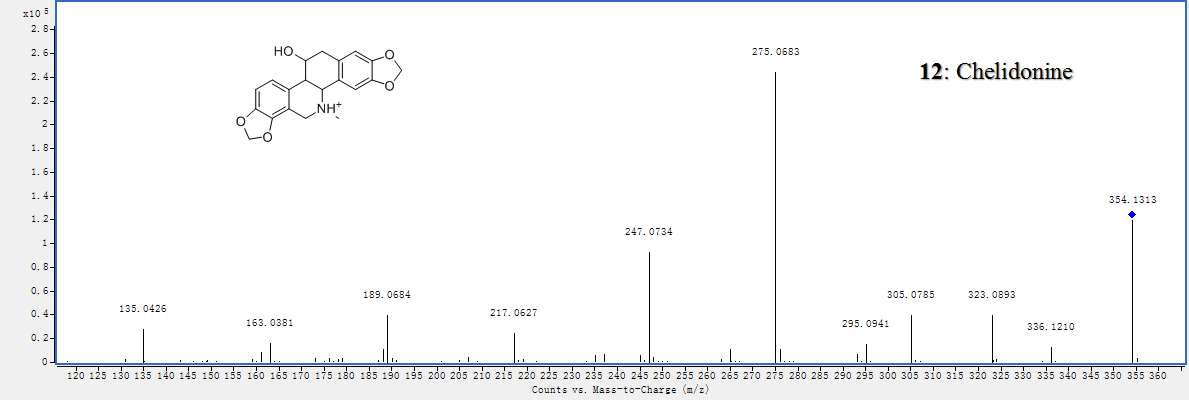


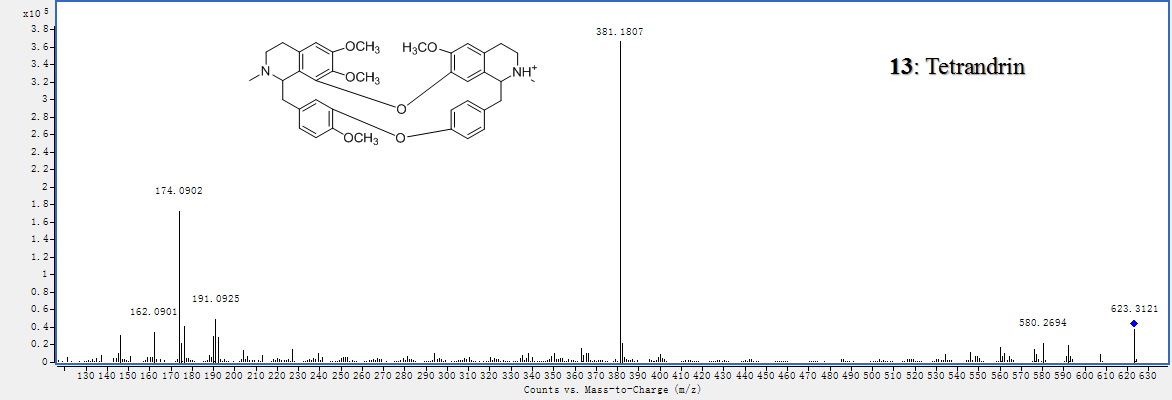


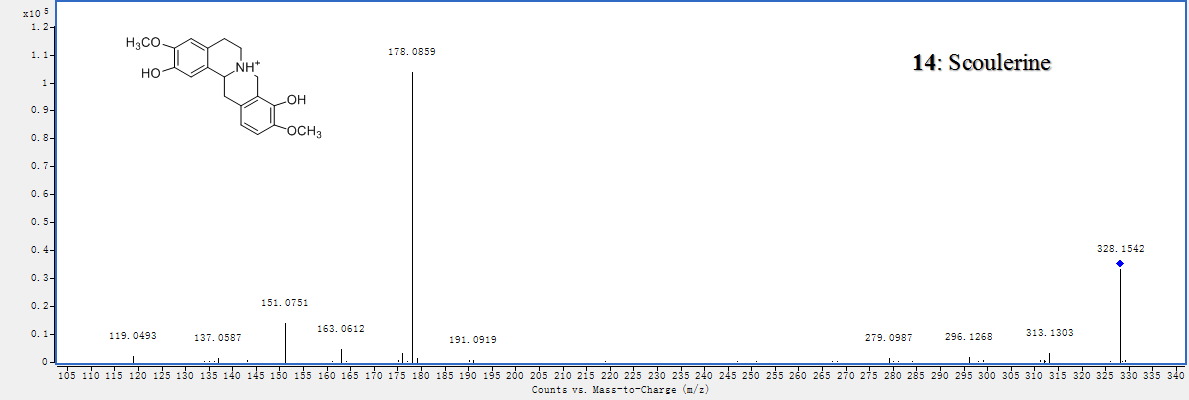


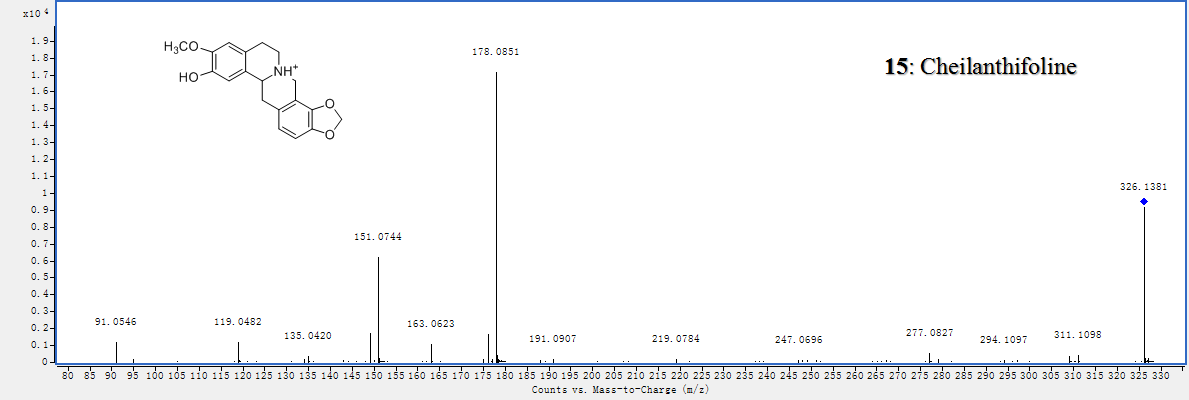


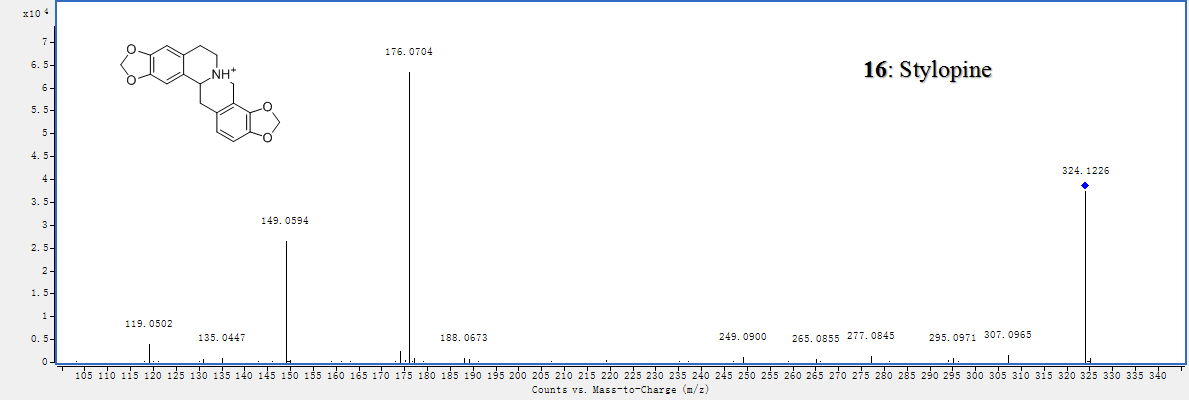


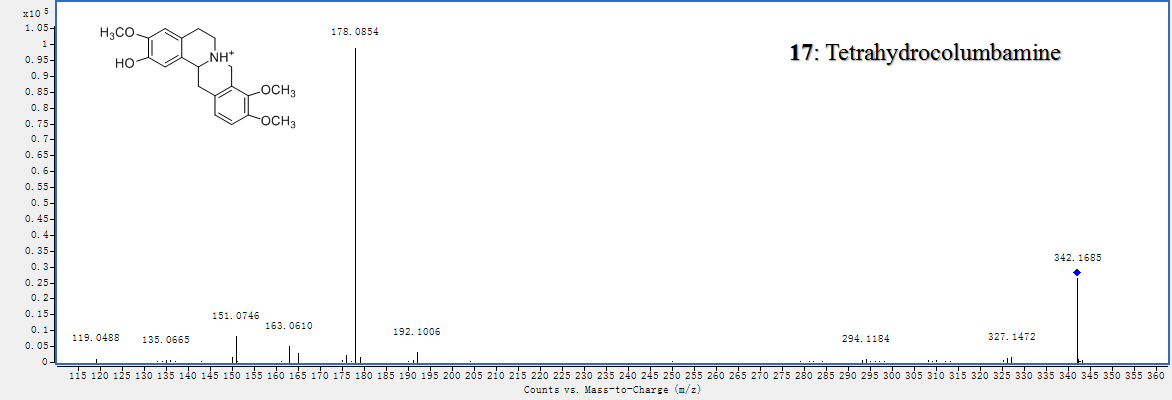


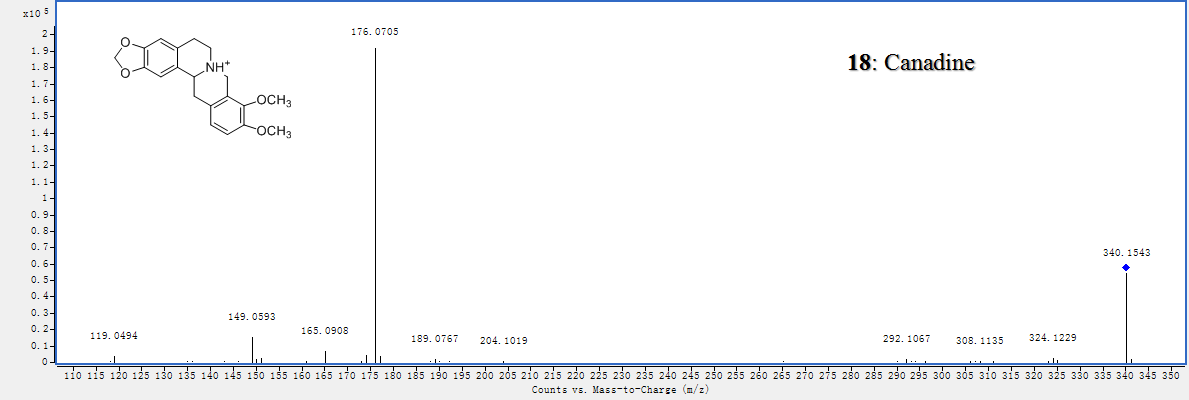


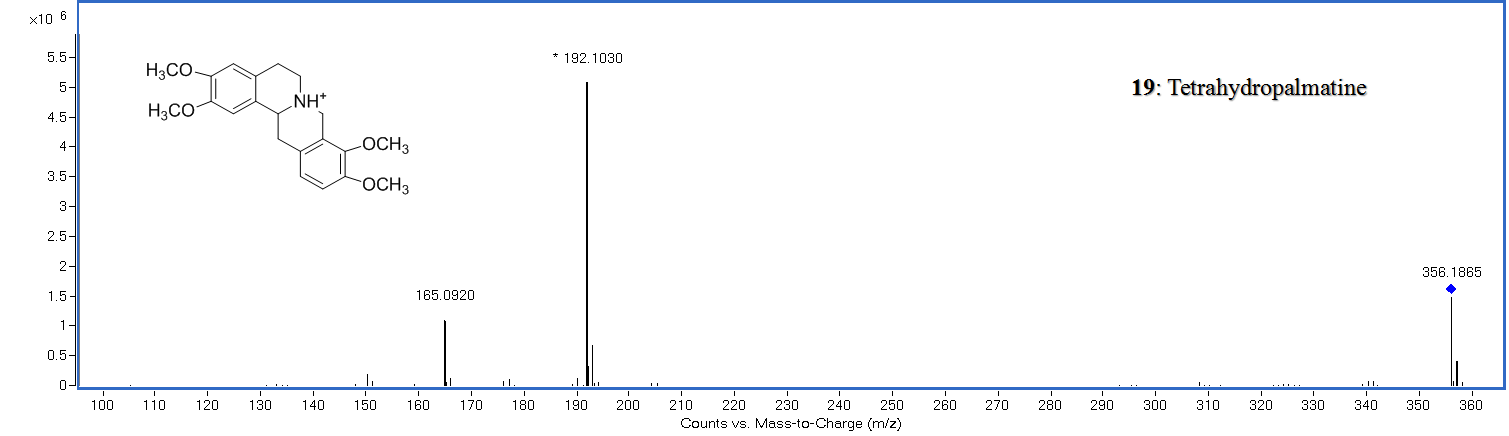


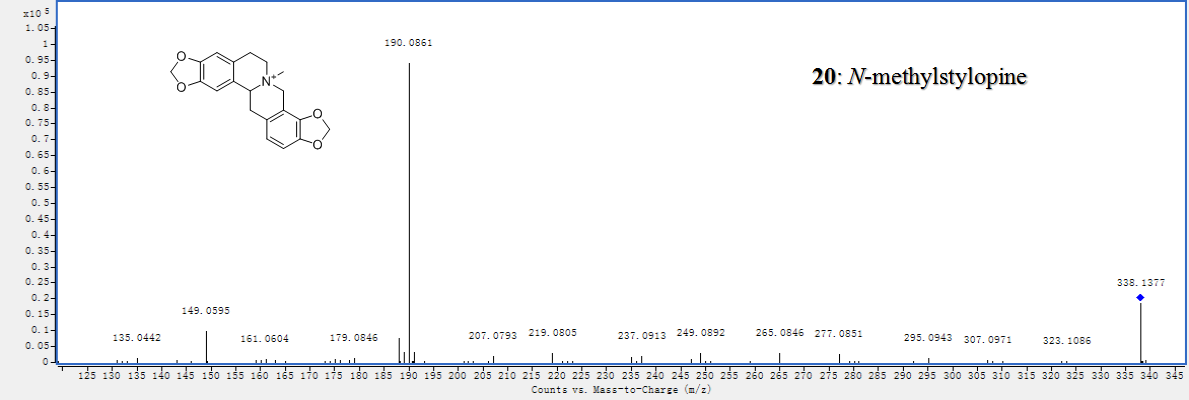


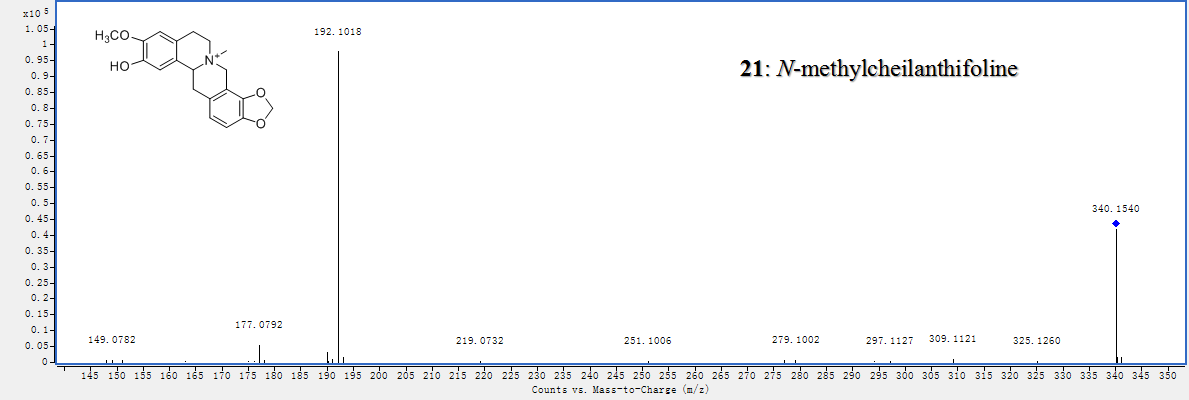


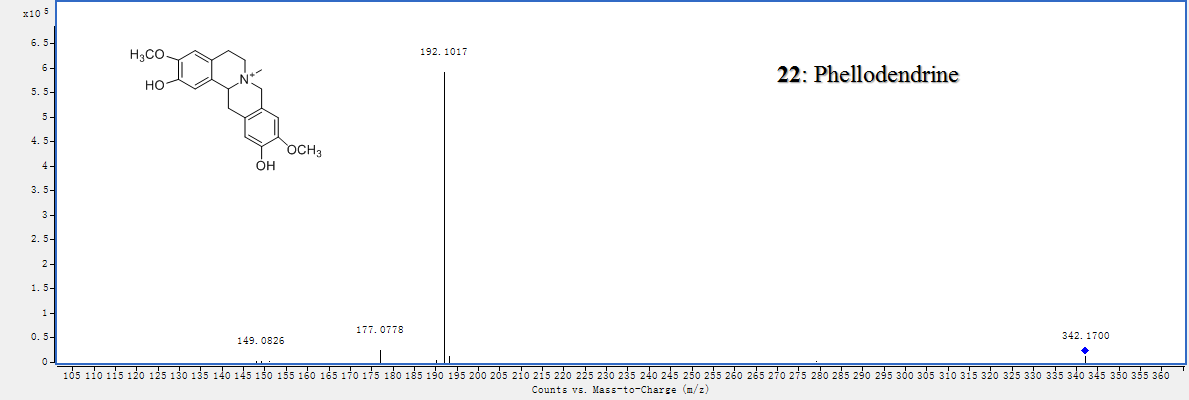


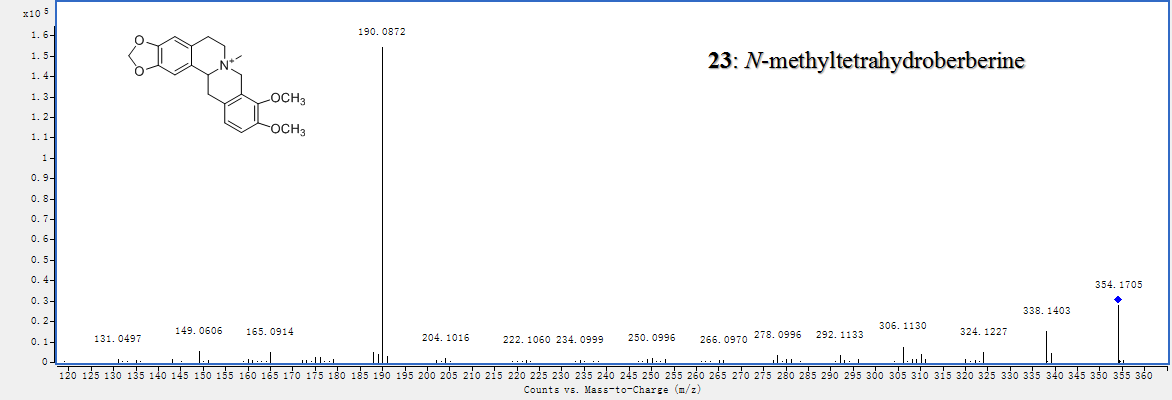


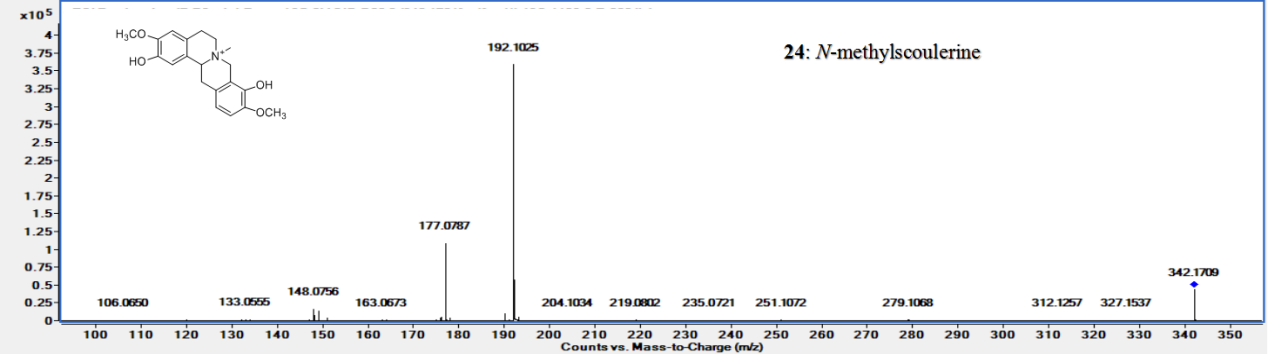


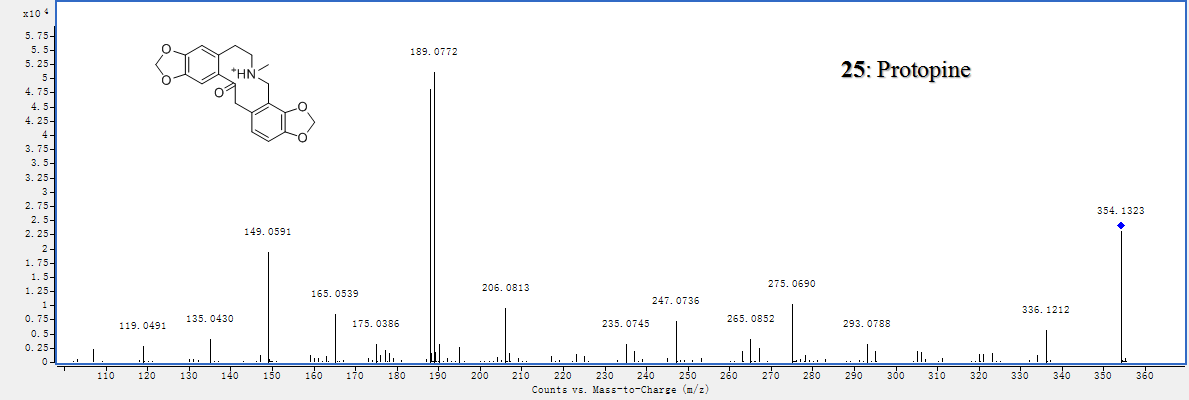


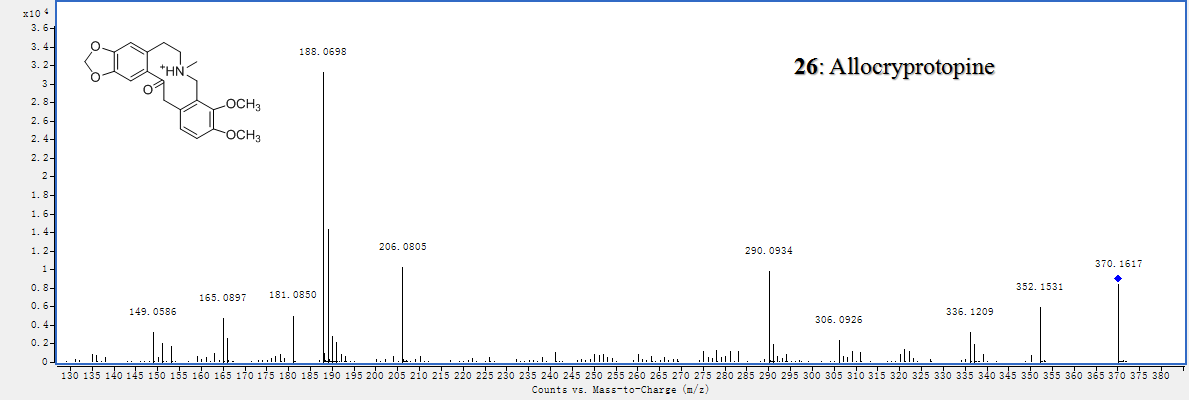


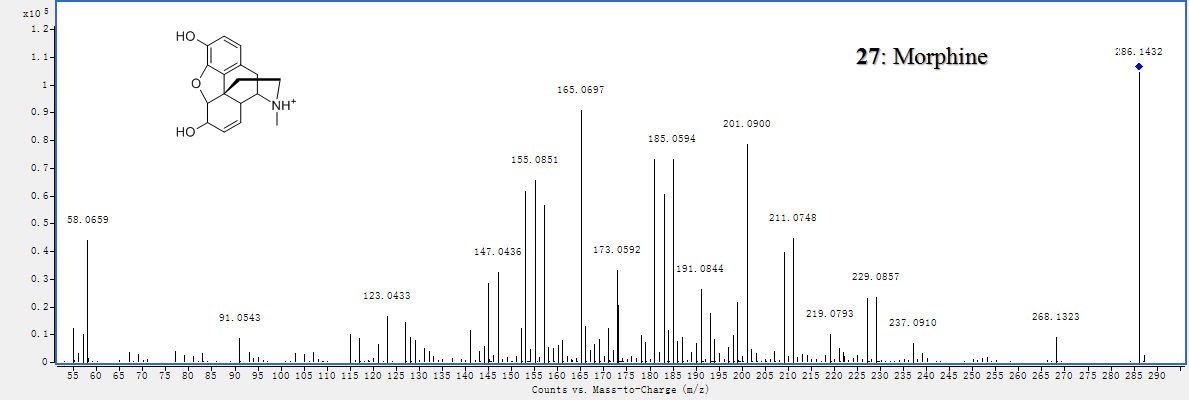


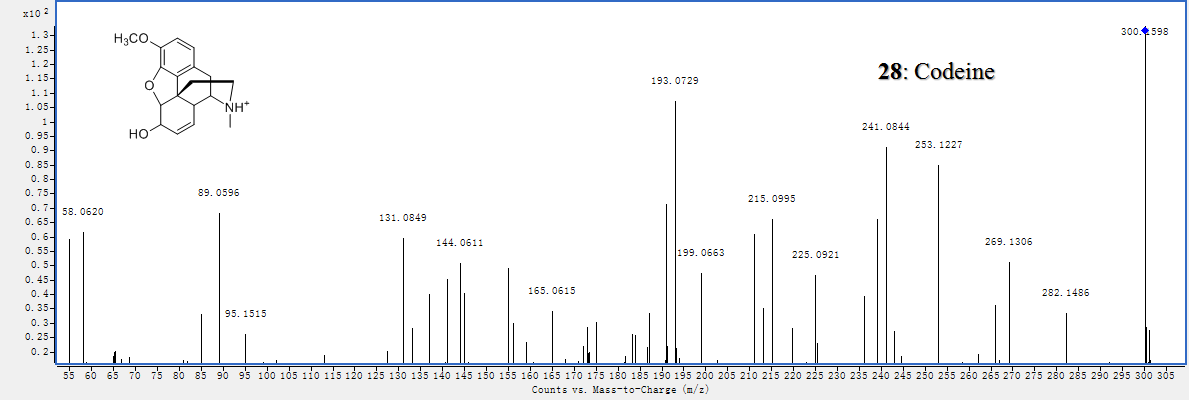


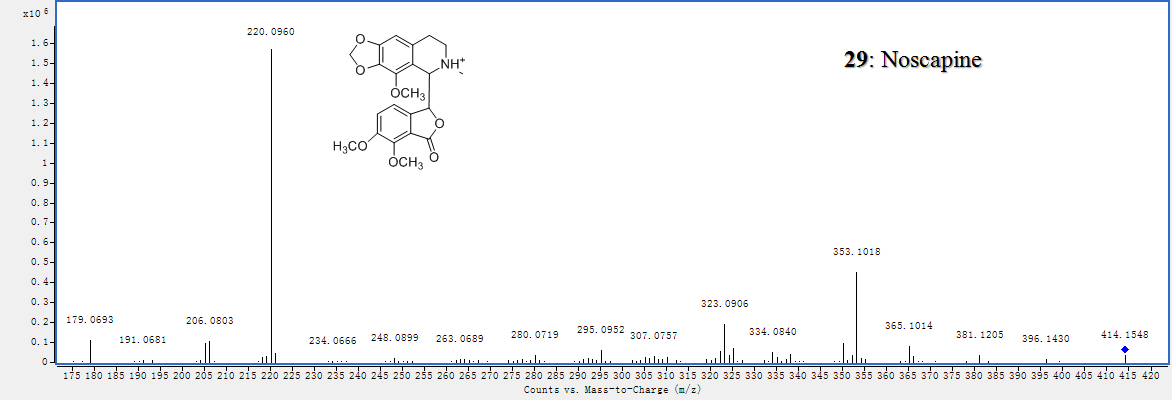


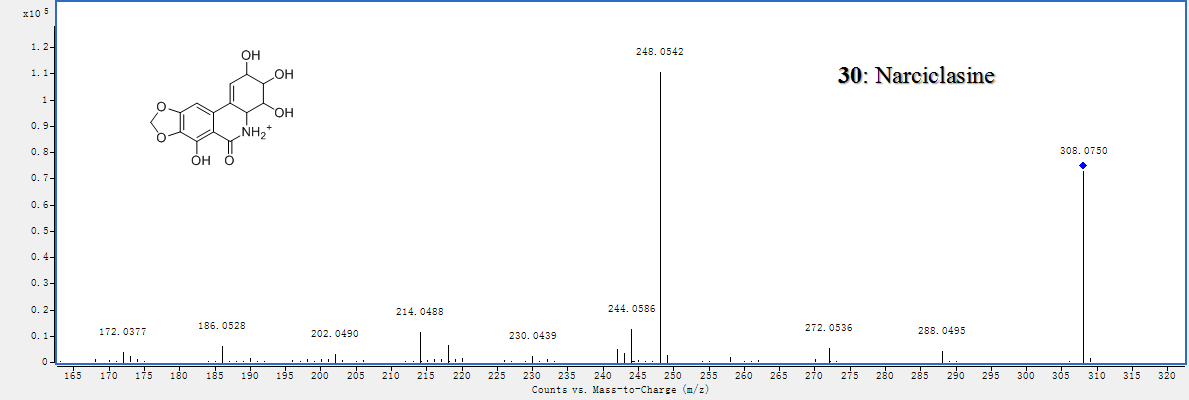


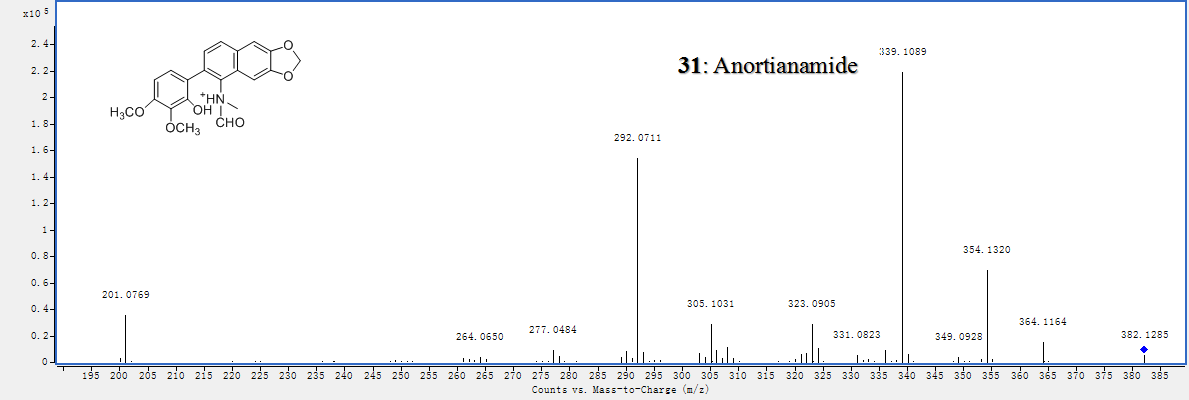


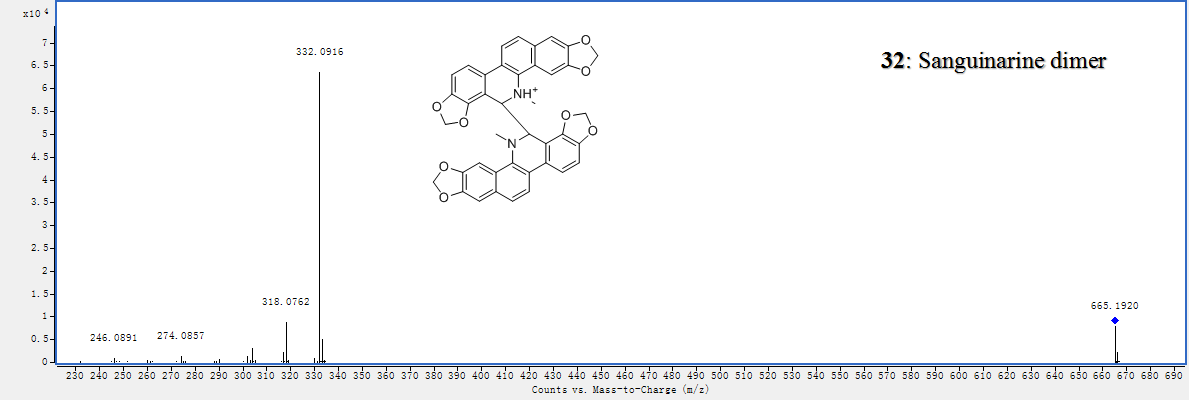


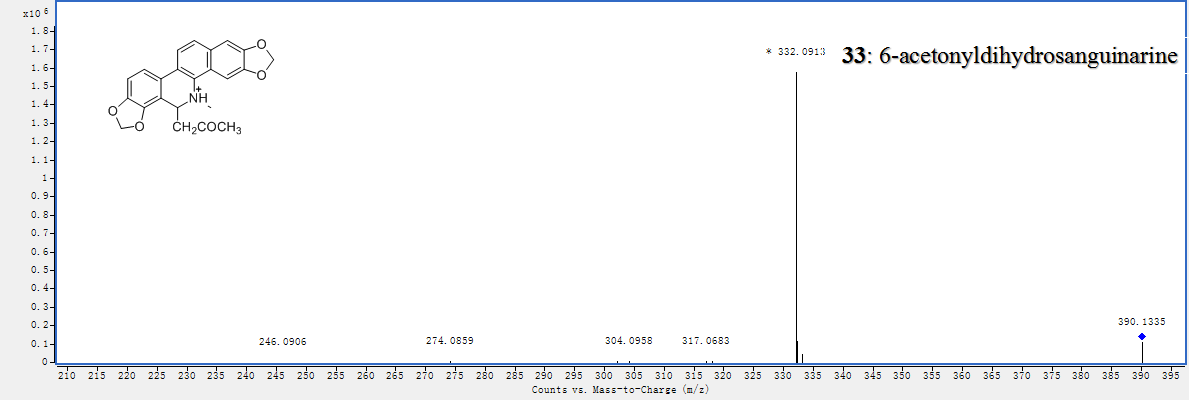


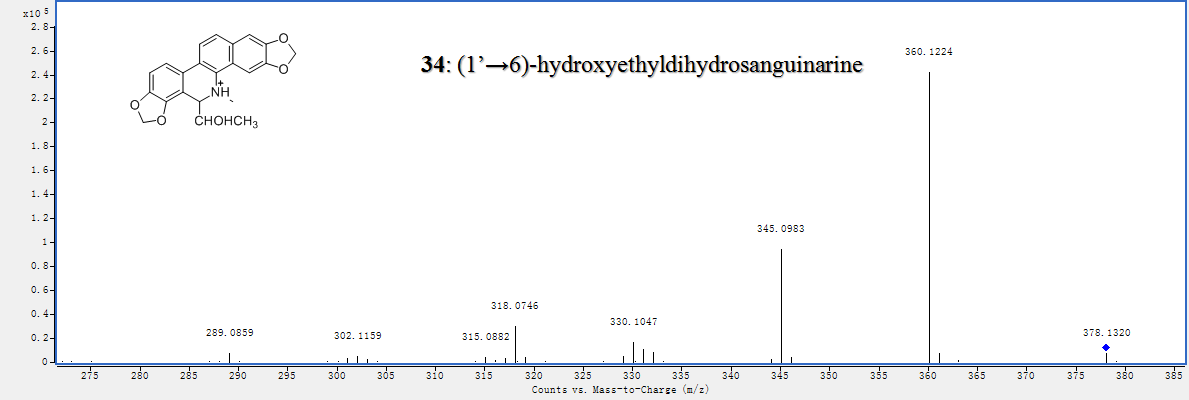


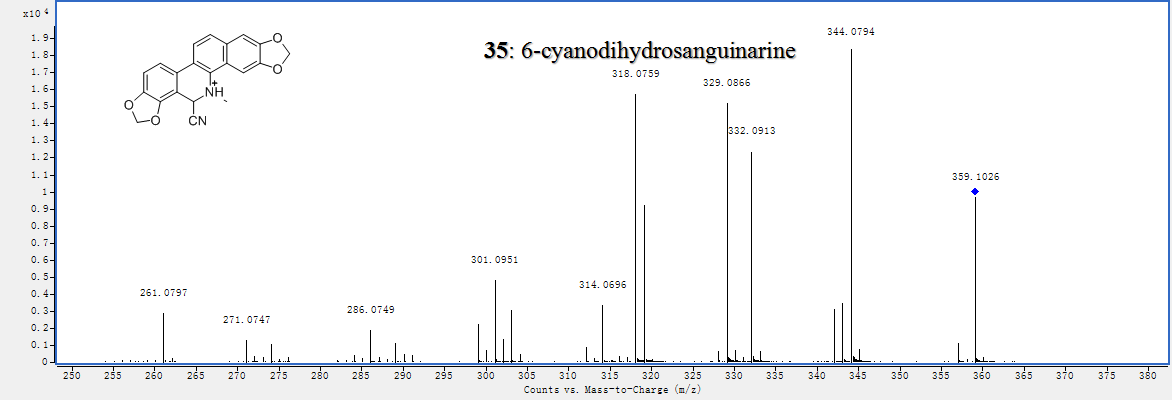


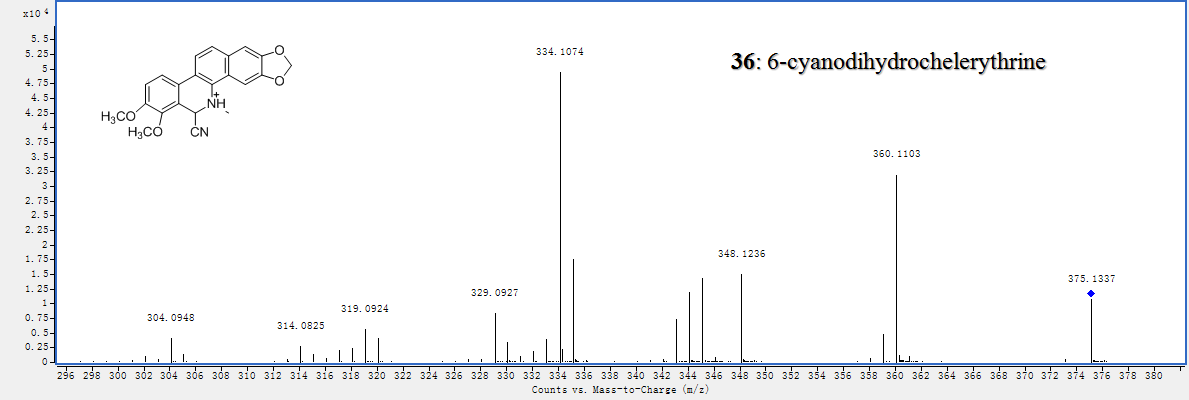


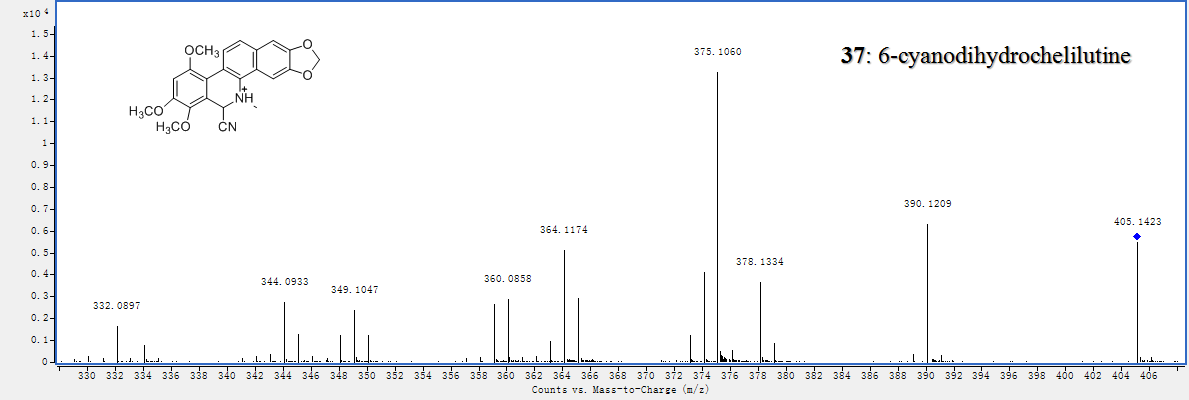


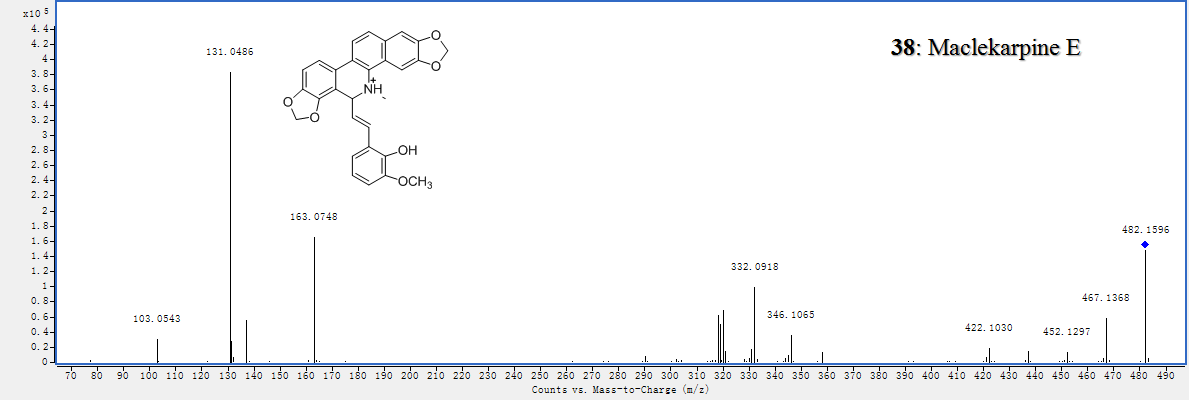


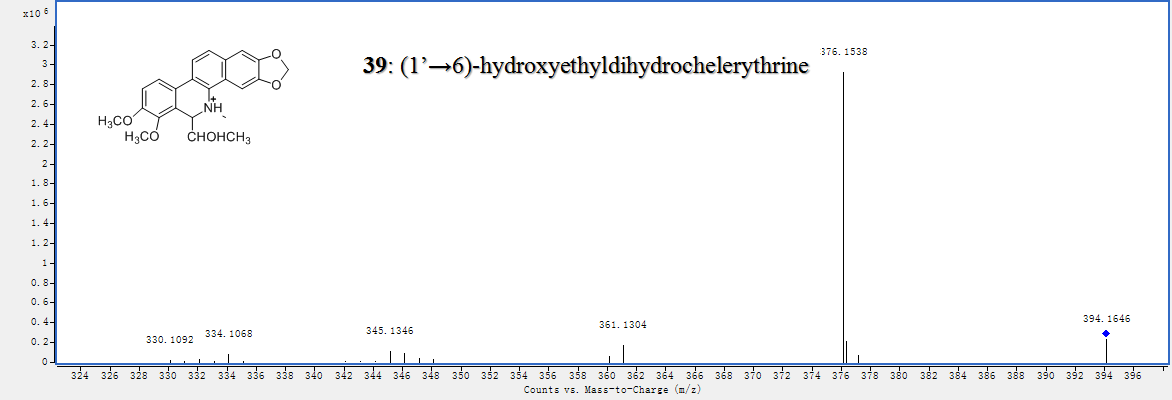


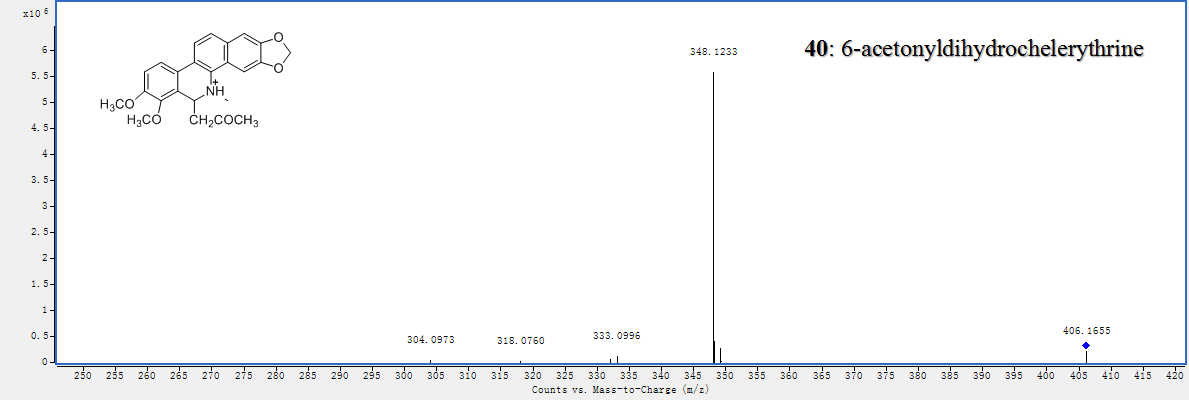


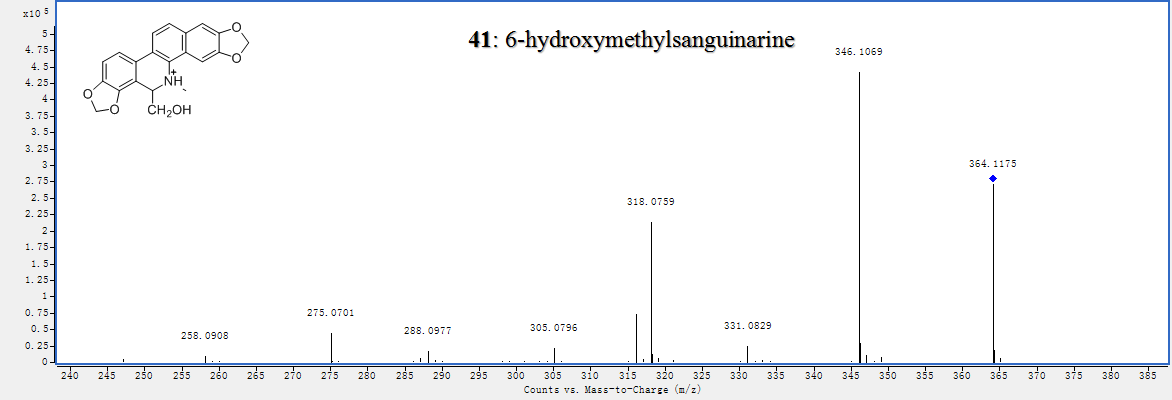


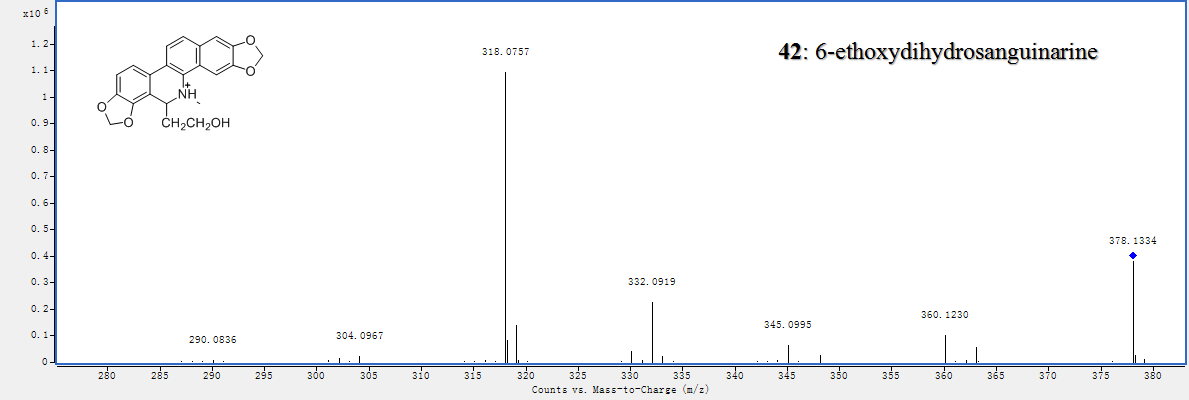


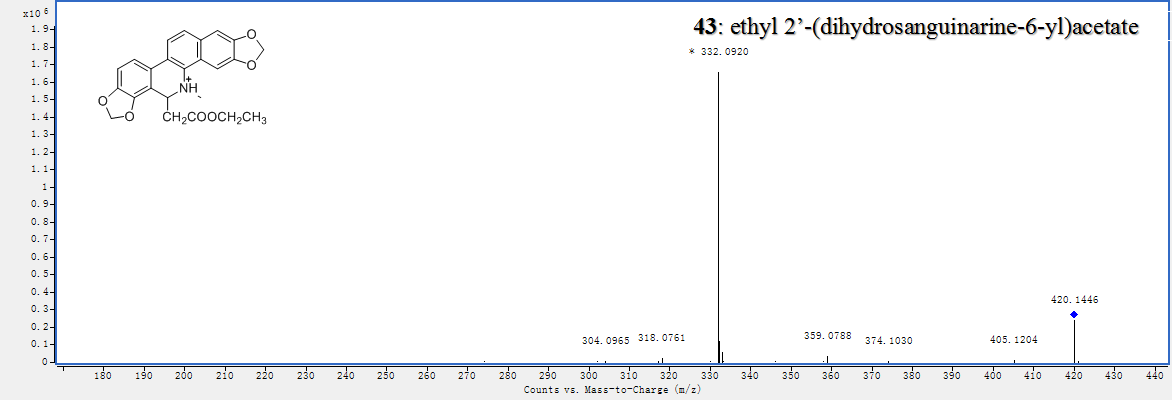


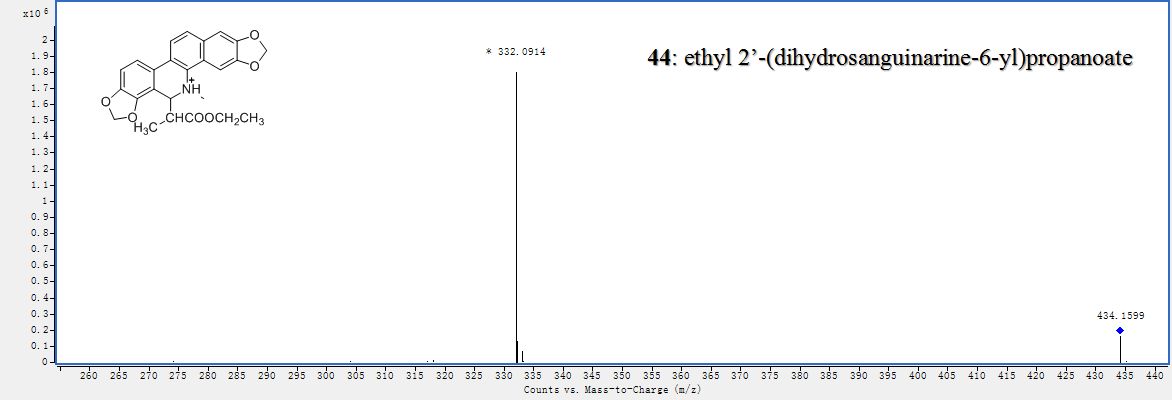


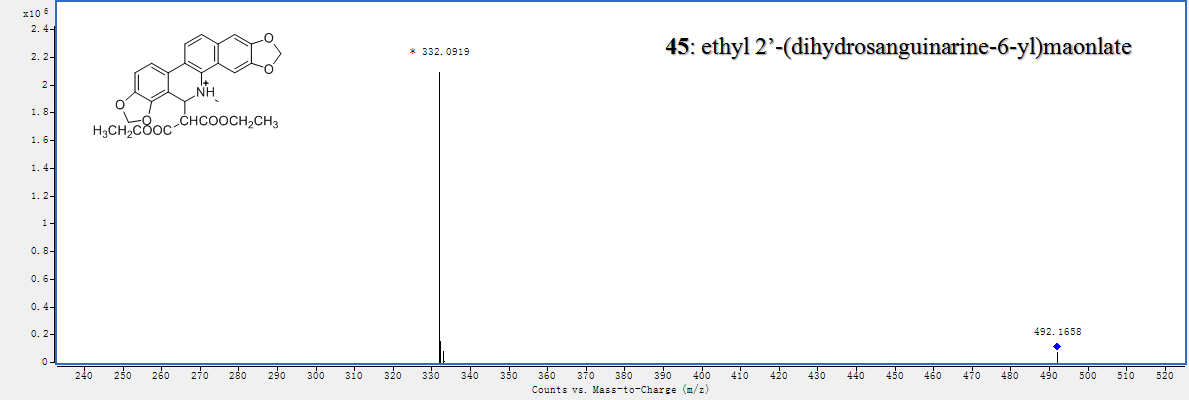


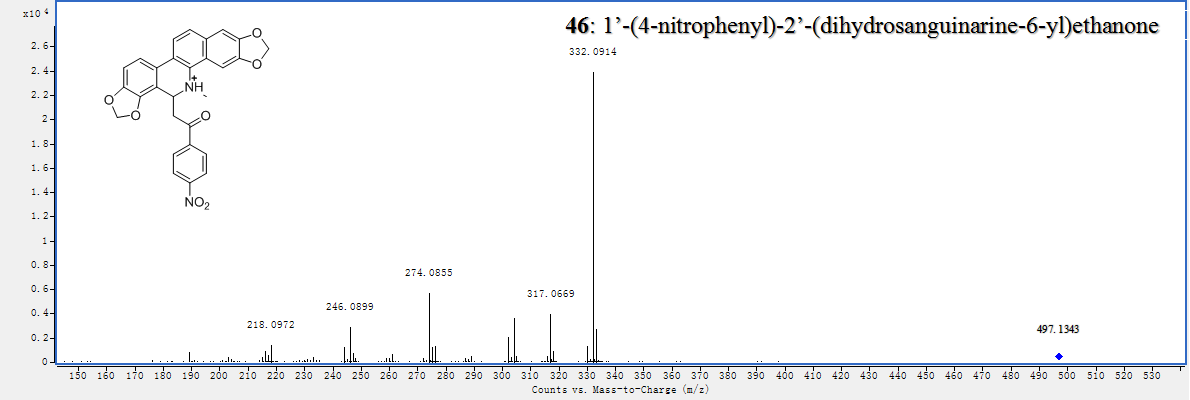


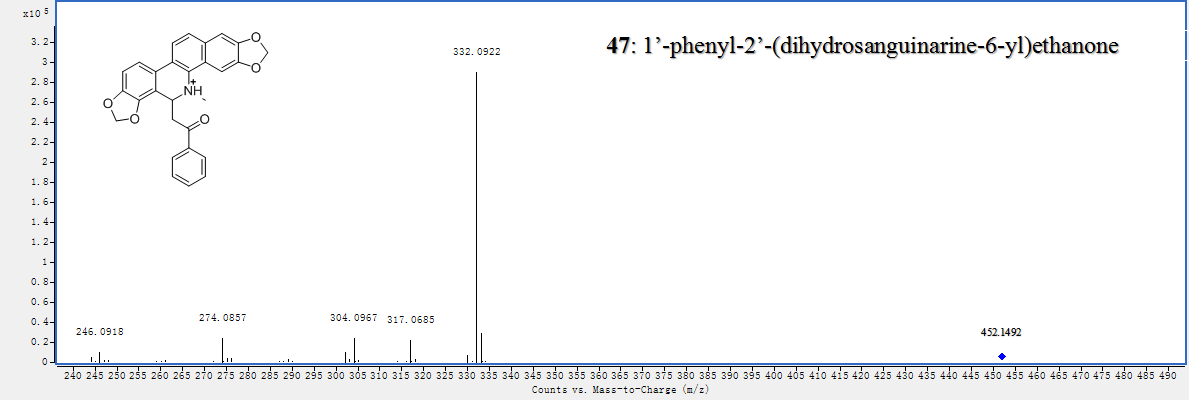


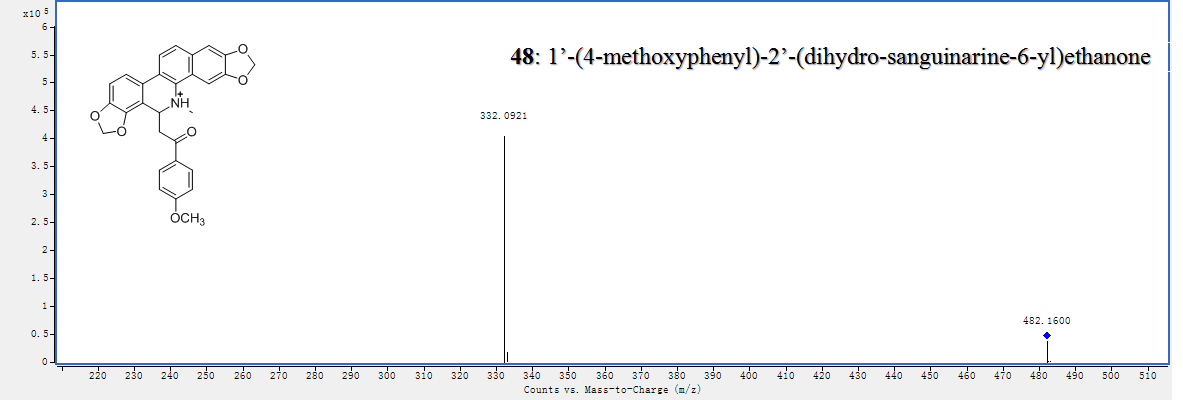


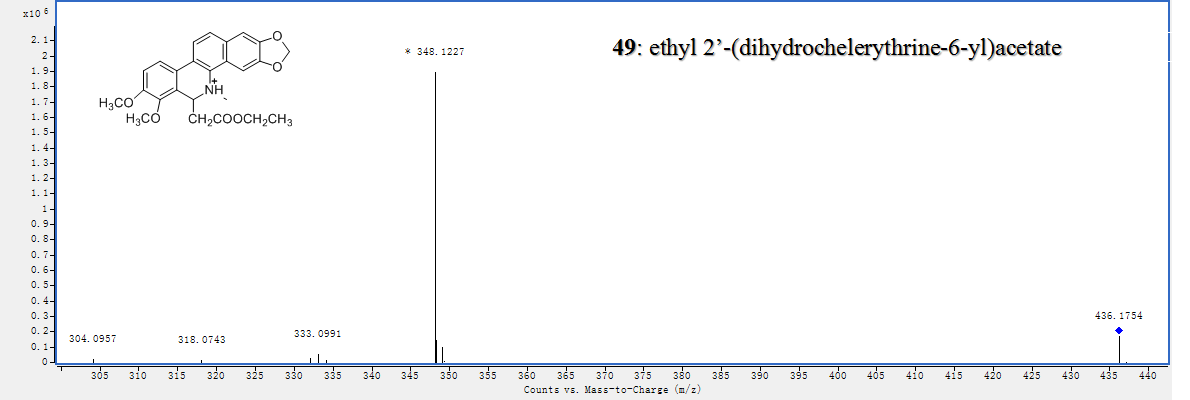


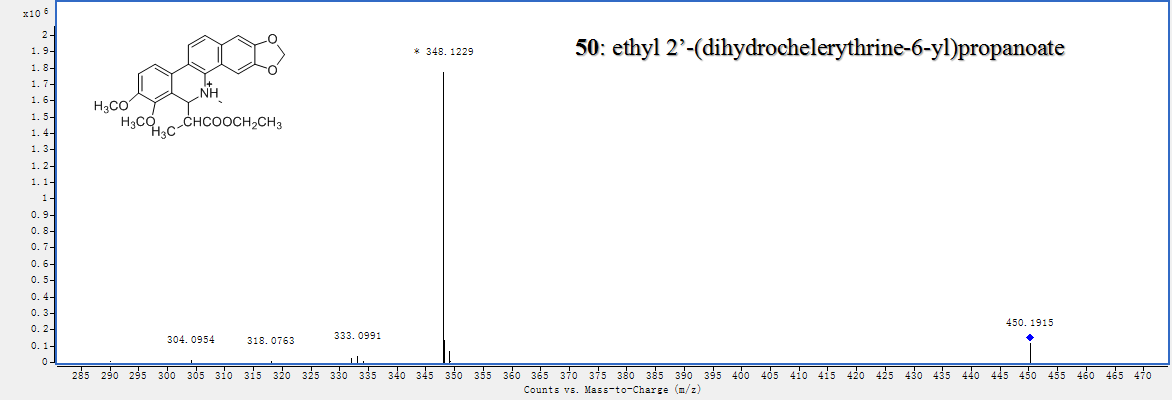


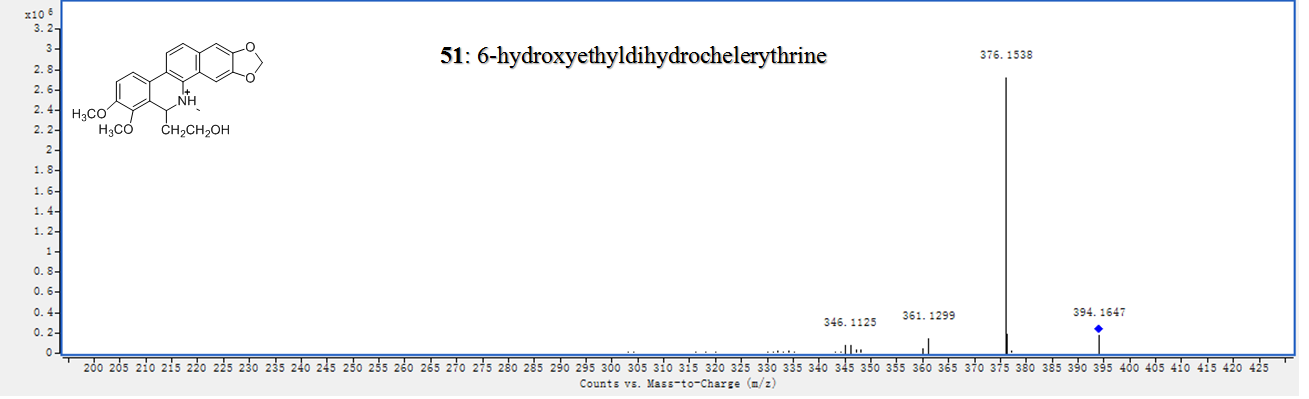


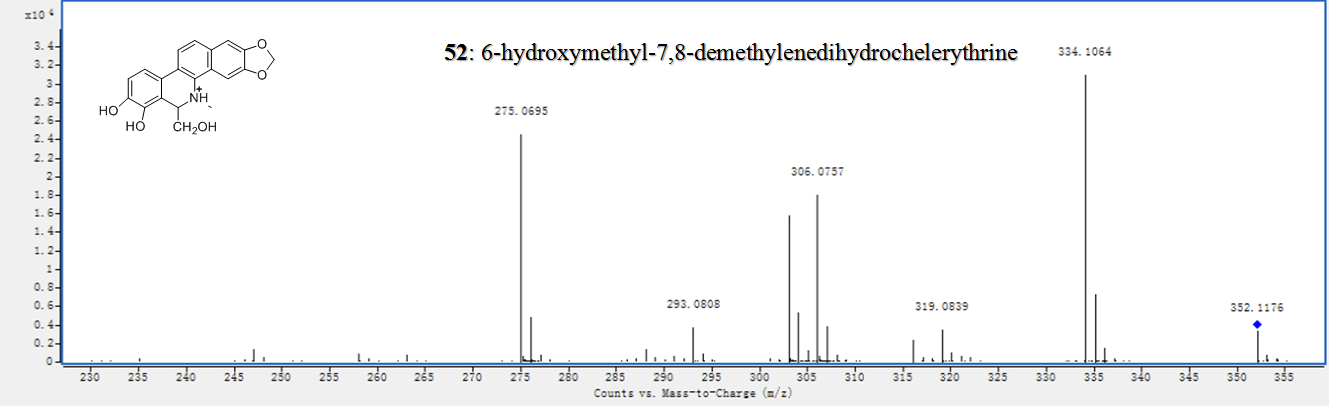


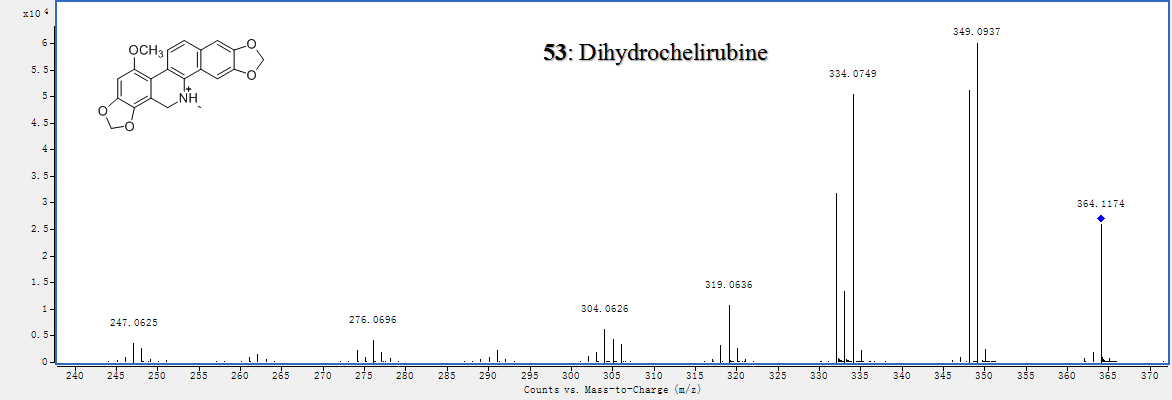


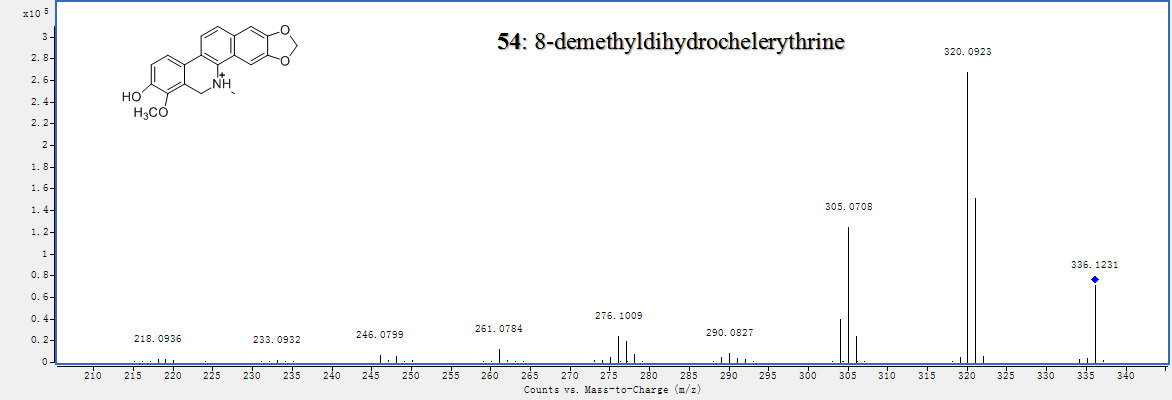


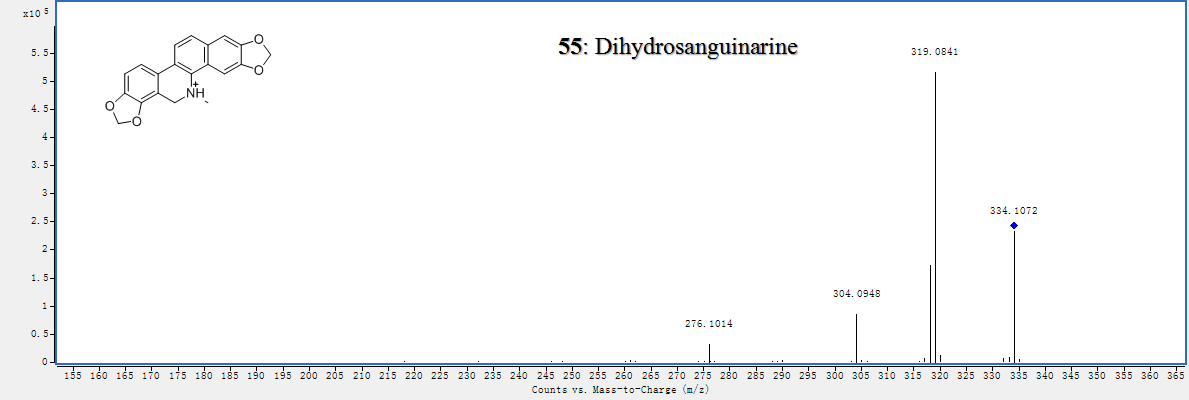


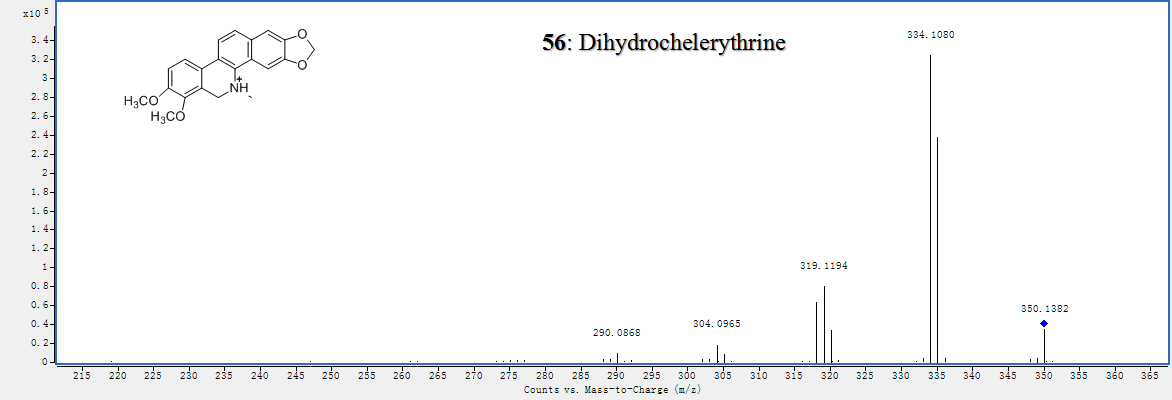


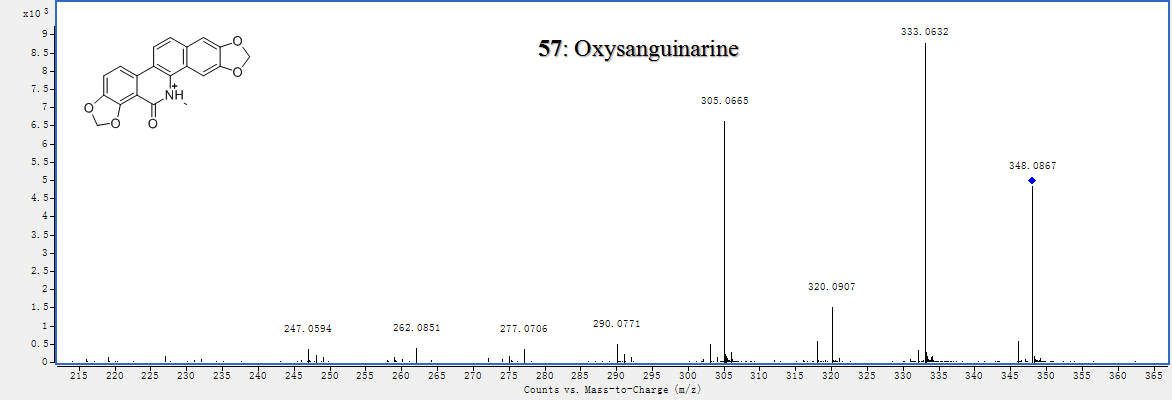


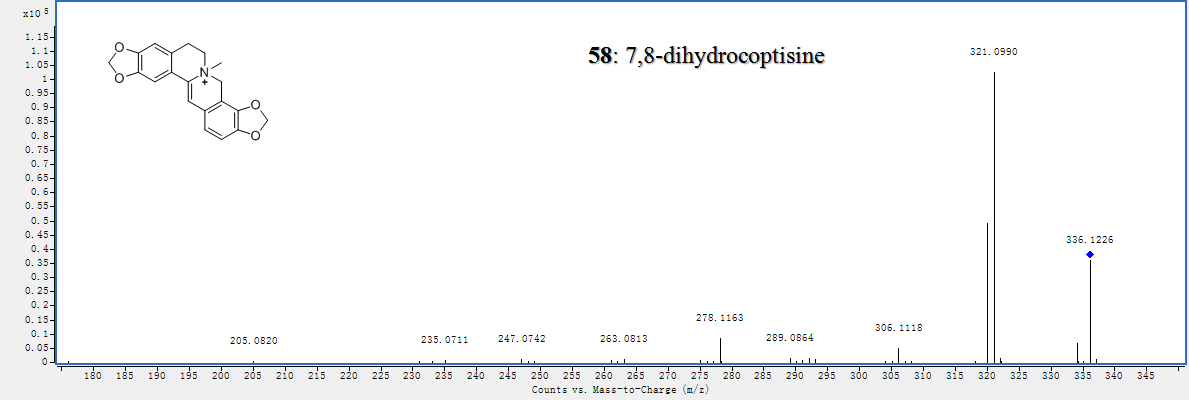


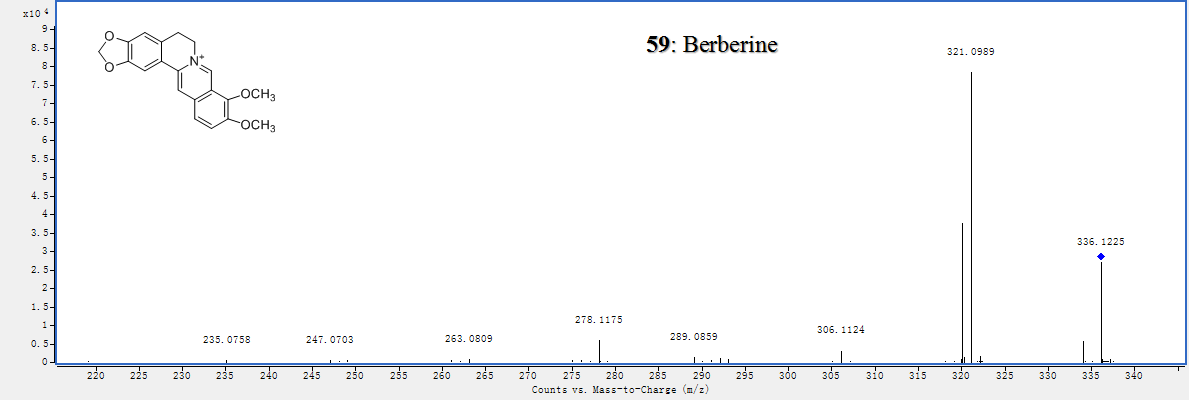


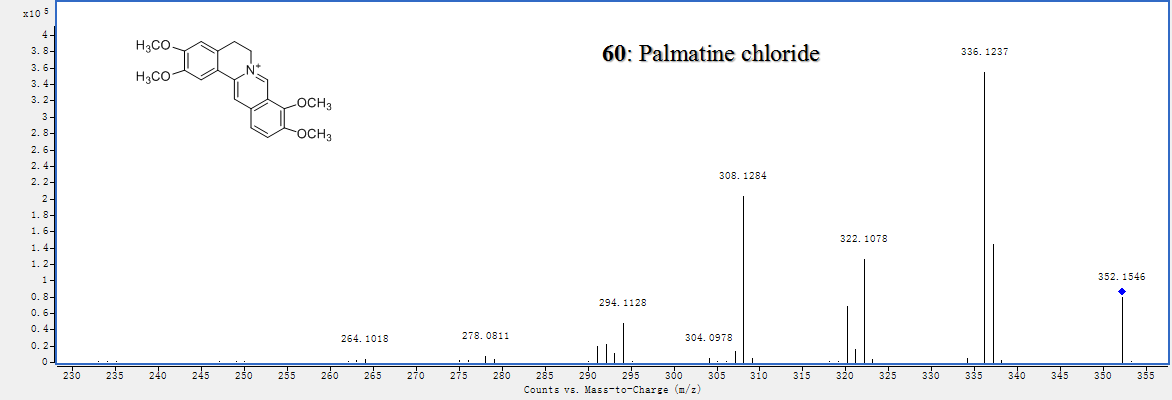


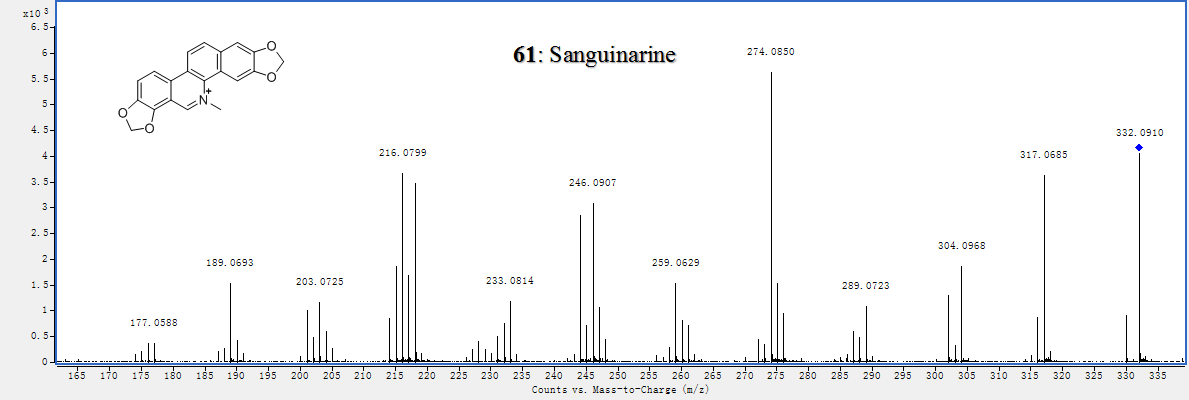


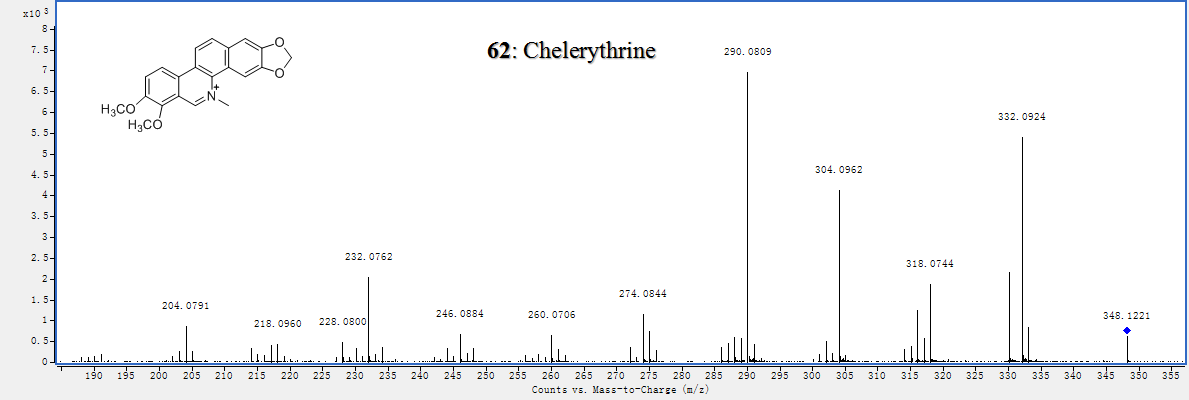


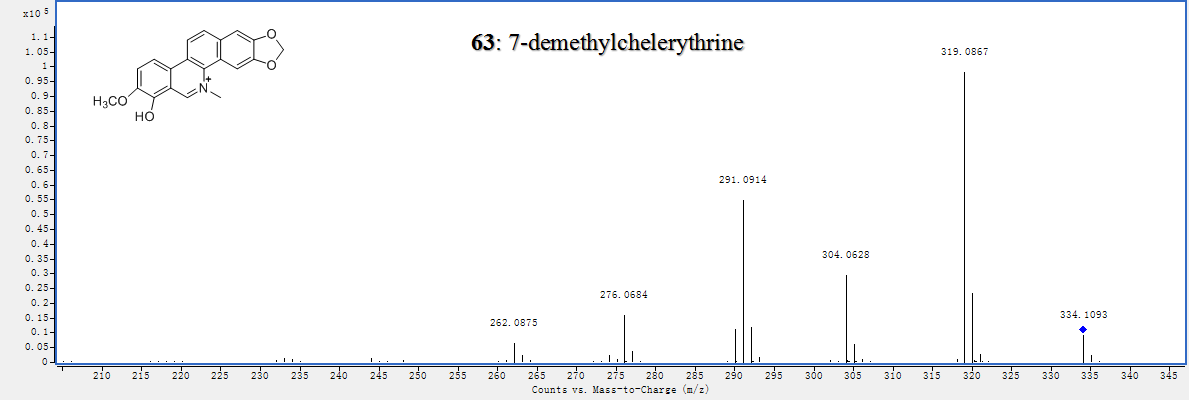


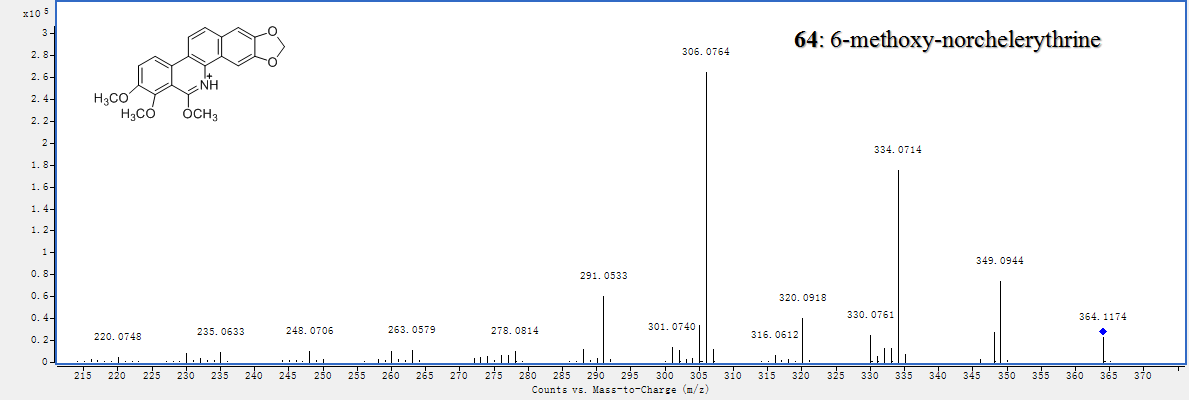


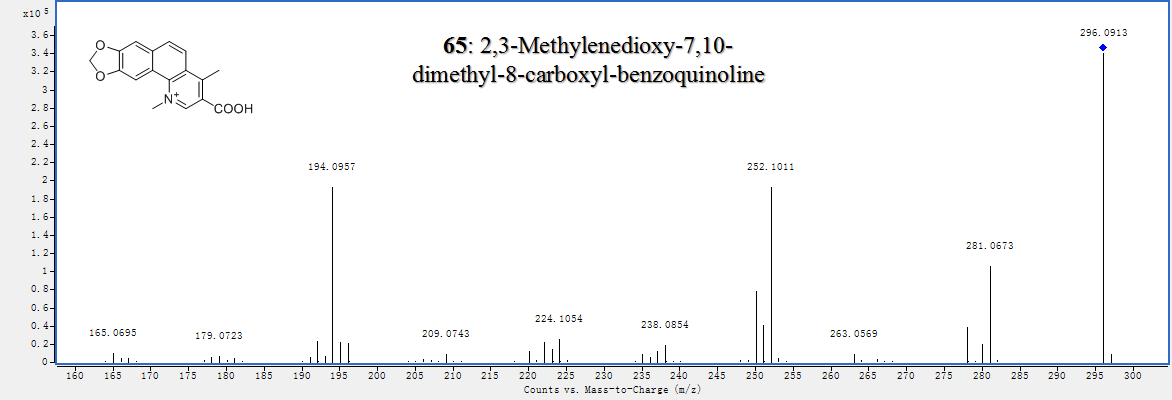


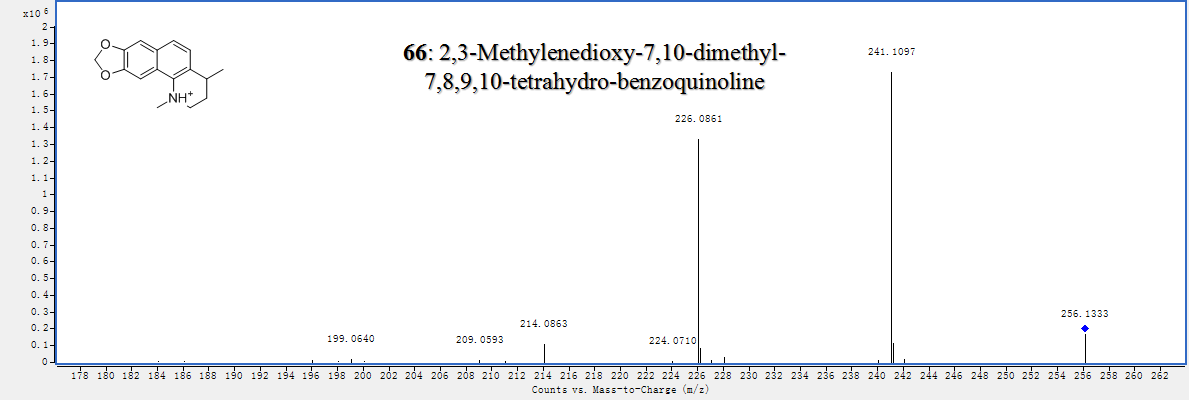


Figure S2 schematic potential energy surface (relative energies were given in kcal/mol) for the proposed [M-NH_3_]^+^ fragmentation reactions of protonated alkaloid **1**, calculated by DFT at RB3LYP/6-31G(*d*) level.


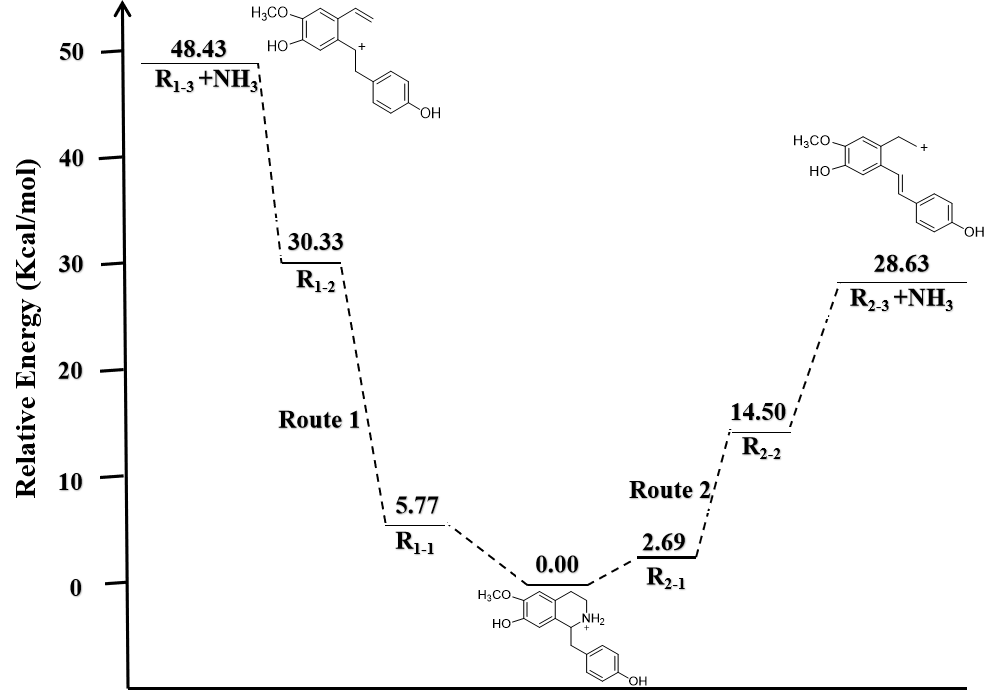

Supplement: Supplementary file 1 — Supplementary Information. [file 41598_2019_57406_MOESM1_ESM.docx]
